# Nano-Theranostics for the Sensing, Imaging and Therapy of Prostate Cancers

**DOI:** 10.3389/fchem.2022.830133

**Published:** 2022-04-12

**Authors:** David G. Calatayud, Sotia Neophytou, Eleni Nicodemou, S. Giuseppe Giuffrida, Haobo Ge, Sofia I. Pascu

**Affiliations:** ^1^ Department of Chemistry, University of Bath, Bath, United Kingdom; ^2^ Department of Electroceramics, Instituto de Ceramica y Vidrio - CSIC, Madrid, Spain; ^3^ Centre of Therapeutic Innovations, University of Bath, Bath, United Kingdom

**Keywords:** nanomedicine, theranostics, sensing, imaging, therapy, prostate cancer

## Abstract

We highlight hereby recent developments in the emerging field of theranostics, which encompasses the combination of therapeutics and diagnostics in a single entity aimed for an early-stage diagnosis, image-guided therapy as well as evaluation of therapeutic outcomes of relevance to prostate cancer (PCa). Prostate cancer is one of the most common malignancies in men and a frequent cause of male cancer death. As such, this overview is concerned with recent developments in imaging and sensing of relevance to prostate cancer diagnosis and therapeutic monitoring. A major advantage for the effective treatment of PCa is an early diagnosis that would provide information for an appropriate treatment. Several imaging techniques are being developed to diagnose and monitor different stages of cancer in general, and patient stratification is particularly relevant for PCa. Hybrid imaging techniques applicable for diagnosis combine complementary structural and morphological information to enhance resolution and sensitivity of imaging. The focus of this review is to sum up some of the most recent advances in the nanotechnological approaches to the sensing and treatment of prostate cancer (PCa). Targeted imaging using nanoparticles, radiotracers and biomarkers could result to a more specialised and personalised diagnosis and treatment of PCa. A myriad of reports has been published literature proposing methods to detect and treat PCa using nanoparticles but the number of techniques approved for clinical use is relatively small. Another facet of this report is on reviewing aspects of the role of functional nanoparticles in multimodality imaging therapy considering recent developments in simultaneous PET-MRI (Positron Emission Tomography-Magnetic Resonance Imaging) coupled with optical imaging *in vitro* and *in vivo*, whilst highlighting feasible case studies that hold promise for the next generation of dual modality medical imaging of PCa. It is envisaged that progress in the field of imaging and sensing domains, taken together, could benefit from the biomedical implementation of new synthetic platforms such as metal complexes and functional materials supported on organic molecular species, which can be conjugated to targeting biomolecules and encompass adaptable and versatile molecular architectures. Furthermore, we include hereby an overview of aspects of biosensing methods aimed to tackle PCa: prostate biomarkers such as Prostate Specific Antigen (PSA) have been incorporated into synthetic platforms and explored in the context of sensing and imaging applications in preclinical investigations for the early detection of PCa. Finally, some of the societal concerns around nanotechnology being used for the detection of PCa are considered and addressed together with the concerns about the toxicity of nanoparticles–these were aspects of recent lively debates that currently hamper the clinical advancements of nano-theranostics. The publications survey conducted for this review includes, to the best of our knowledge, some of the most recent relevant literature examples from the state-of-the-art. Highlighting these advances would be of interest to the biomedical research community aiming to advance the application of theranostics particularly in PCa diagnosis and treatment, but also to those interested in the development of new probes and methodologies for the simultaneous imaging and therapy monitoring employed for PCa targeting.

## 1 Introduction

Non-communicable diseases (NCDs) are a group of medical conditions or diseases which are not infectious or transmissible. Autoimmune diseases, heart diseases, strokes, cancers, diabetes, chronic kidney disease, osteoporosis, Alzheimer’s disease and cataracts are the most common NCDs spread worldwide. In many cases, they are referred to as “chronic diseases” for their long periods of persistence and slow progression which can also lead to death (Daar et al., 2007). Nowadays, NCDs kill 41 million people worldwide (World Health Organization, 2021), equivalent to 71% of all deaths globally, and therefore represent one of the most important causes of premature death.

Recent studies have shown that the NCDs are associated with risk factors, attributed to behavioral models (Hunter and Reddy, 2013; Singh et al., 2018). The most common risk factors found in the studies during these last 20 years are unhealthy diet, physical inactivity (Forouzanfar et al., 2016), alcohol (Parry et al., 2011), and tobacco (Hill and Peters, 1998). Lifestyle choices including alcohol consumption and smoking have been highlighted as causes for several different health conditions such as liver cirrhosis, cardiovascular disease, stroke, hypertension and some forms of cancer (World Health Organization, 2021). These four main risk factors of NCDs have in common the onset of cancer. Among the different of cancer, Prostate cancer (PCa) is the second most common type worldwide. This malignanctypesy counted about 1.3 million new cases and caused around 360,000 deaths in 2018 (Rawla, 2019).

Prostate cancer is the most commonly diagnosed cancer in males in the United Kingdom, with more than 47,500 patients diagnosed with this cancer every year: in this sense, 129 men every day would benefit from earlier detection and diagnosis as every 45 min one man dies from prostate cancer, i.e., more than 11,500 men every year Cancer Statistics, 2021. According to the charity Prostate *Cancer* United Kingdom, 1 in 8 men will be diagnosed with prostate cancer in their lifetime and around 400,000 men are living with and beyond prostate cancer (Source: Prostate *Cancer* United Kingdom). Monitoring the effect of treatment, as well as engaging in recurrence prevention would benefit from simple and non-invasive diagnostics and monitoring tools, much the same as it is currently the case with other non-communicable diseases which are treated in communities, e.g., diabetes. Therefore, there is an increased clinical drive to adopt personalized treatment strategies to reduce patient exposure to, and healthcare expenditure on, unnecessary treatments. The mainstay in diagnosis is the detection of the prostate-specific antigen (PSA), however this test is known to be unreliable at the lower ranges of PSA levels in serum, and prone to providing both false positive and negative readings for PCa. There is an increased clinical drive to adopt personalised treatment strategies to reduce patient exposure to, and healthcare expenditure on, unnecessary treatments.

On average, the 5-years survival rate of patients with localized PCa exceeds 90%. However, patients with distant metastases have significantly lower 5-years survival rates, averaging approximately 31% for prostate cancers (Siegel et al., 2021). In this context, there are limitations regarding the collection of the necessary data as in-population analyses rely on self-reporting or self-evaluation. Other limitations in terms of diagnostics and therapeutics advances are derived from the fact than many theranostics show potential under controlled laboratory settings in animal studies with only limited transferability to human patients.

The major diseases affecting the prostate are prostatitis (inflammation of the prostate due to infection), autoimmune diseases and cancer, an NCD. Almost 75% of the human genome is expressed in prostate cells, yet several genes are highly prostate-specific. These are the prostate specific antigen (PSA), the prostatic acid phosphatase (PAP) or the prostate-specific membrane antigen (PSMA).

To date, The main causes of prostate cancer remain unclear. However, obesity, age and family history are the main risk factors for this disease (Key, 2011). The risk of developing this cancer have been suggested to be related to age: males over 50 years old are common patients with a high risk of developing prostate cancer; rare cases have been found in men under 50. Genetic background related to ethnic, family and specific gene variants could contribute to the risk of the development of prostate cancer. (Steinberg et al., 1990; Edeline et al., 2017; Ledet et al., 2017). Other factors that have been hypothesised to increase the risk of developing prostate cancer (Lane et al., 2017; Peisch et al., 2017), being linked to diet such as excessive consumption of red and processed meat infections with *chlamydia*, gonorrhea, and syphilis (Dennis et al., 2002; Sarma et al., 2006), or presentation with prostatitis. However, many of the factors that were hypotheised to increase or decrease the risk of prostate cancer have not been definitively demonstrated and while most men with early prostate cancer are asymptomatic, the treatments are most effective when the disease is diagnosed early. Once diagnosed, there are a number of treatments for PCa, many with life changing side effects (Prostate *Cancer* United Kingdom, Available from: https://prostatecanceruk.org/prostateinformation/treatments.). It remains the case that access to early diagnosis of prostate cancer (PCa) represents a major barrier to effective treatment.

The annual medical costs for cancer care in the United Kingdom (across the NHS, private and voluntary sector) were estimated at ca. £9.4 billion since 2010, equivalent to an average of ca. £30,000 per person with cancer per year, whereas the equivalent EU costs were estimated at €126 billion. A recent public health review publication estimated a current total cost of cancer of ca. €199 billion in Europe (EU-27, Iceland, Norway, Switzerland, and United Kingdom). Of this total ca. €32 billion are being spent on cancer drugs. Over the past decade, estimations of the cost of PCa nearly doubled worldwide, partly due to an ageing population, whilst the annual economic productivity loss due to cancer was ca. €70 billion in 2018. (Luengo-Fernandez et al., 2013; Hofmarcher et al., 2020).

Therefore, The early diagnosis of prostate cancer is of great importance for patients, as 5-years survival and remission rates drop rapidly for late-stage diseases. While patients who have been diagnosed early have a survival chance of over 95%, 5-years survival rates for patients with local or inter-organ metastases drop to ca. 50 and under 30%, respectively. Most early-stage prostate cancers are not diagnosed sufficiently early likely due to lack of symptoms, ambiguity thereof, and an absence of suitable in-population screening methods that are widely accessible geographically as well as in terms of patient acceptability. The primary neoplastic growths in PCa are often asymptomatic, ambiguous and as such often denoted “silent killers”. Since these are frequently mis-diagnosed, or diagnosed late, screening of at-risk groups is of great importance for PCa, however no reliable screening method suitable for in-population settings has been developed so far.

PCa can be detected with preliminary tests such as DRE test (digital rectal examination): a tactile method used by doctors to find abnormal parts on the prostate and, at last, diagnose occurring cancer. Also, different substances can be produced in response to cancer and can be used as biomarkers for detecting prostate cancer in an early stage. For instance, the PSA (prostate-specific antigen) test is used to measure the level of the enzyme PSA in male blood. Usually, a high level of PSA may indicate a risk of PCa and doctors recommend regular tests over time to evaluate if a biopsy is needed. Similarly, 4Kscore and the PHI (prostate health index) are other tests (Eastham, 2017) used to evaluate the developing of prostate cancer. Unfortunately, these diagnosis methods can lack sensitivity and specificity, resulting in false-positive or negative responses, and can be invasive for patience. Imaging and biosensing methodologies emerged over the past 2 decades as powerful tools to detect and localise prostate cancer cells at both early, and late, stages of the disease.

## 2 Bioimaging Methods and Biosensing Tools for Detecting Prostate *Cancer*

**SCHEME 1:**
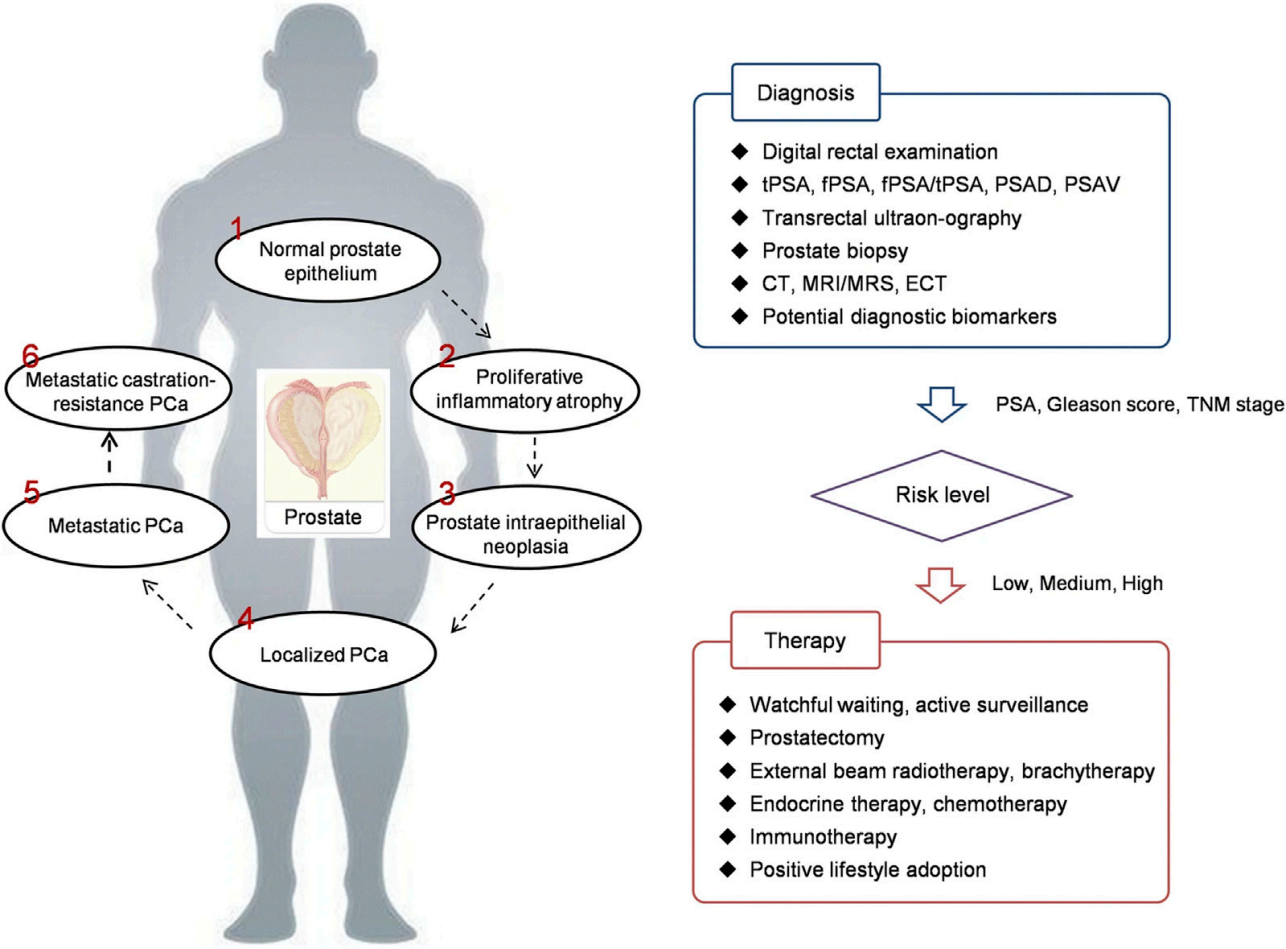
PCa development and theranostics. PCa prostate cancer, PSA prostate speciﬁc antigen, tPSA total PSA, fPSA free PSA, PSAD PSA density, PSAV PSA velocity, CT computed tomography, MRI magnetic resonance imaging, MRS magnetic resonance spectroscopy, ECT emission computed tomography, TNM tumor node metastasis (Lin et al., 2020).

Usually, there are no clear symptoms in early stage PCa. Only when the tumor is enoght big, symptons appear in the lower urinary tract when the urethra and bladder have been affected. There are a series of clinical strategies can be used for PCa detection, including digital rectal examination (DRE), PSA testing, transrectal ultrasonography, CT, emission computed tomography (ECT), and MRI (Scheme 1) (Lin et al., 2020).

The mainstay in detection relies on the widely used and minimally invasive screening of levels of the prostate specific antigen (PSA) in serum. However, this method has seen decreased usage over the last few years, due to its low accuracy. PSA is an enzyme produced and secreted by a certain level by prostate cells; all men have a certain level of this protein in their serum although under certain conditions this can be overexpressed as show elevated levels in blood serum. While certain cancers overexpress PSA, other conditions may also raise the PSA levels, for example, infection or benign prostatic hyperplasia. There are wide variations in the PSA levels within individuals, for example due to age or ethnicity. As such, the PSA levels can only be used reliably for a given patient in case a ‘baseline’ is already available for that patient. The National Health Service (NHS) United Kingdom does not regularly perform PSA screening on this basis and it has been argued that the majority of procedures carried out due to the as-found high raised PSA levels could be unnecessary and potentially harmful.

Achieving early diagnosis through effective screening could lead to impact for patients that may be significant. Howeevet, as mentioned the value of PSA as the biomarker of choice in determining the extent and consequences of the disease is under discussion. This is due to characteristic indolence of the tumours, the high level of PSA related to prostatic anomalies, culminating in over-diagnosis and unnecessary biopsies affecting the patient’s quality of life (Ahmed et al., 2017).

Current state-of-the-art in PCa screening is through biosensing assays. This method relies on detection of the prostate-specific antigen (PSA) by enzyme-linked immunosorbent assays (ELISA) in a patient’s serum. However, these methods struggle to achieve detection limits of the order of several ng/ml of PSA as the receptor of choice. Higher sensitivity is urgently needed to enable detection of the biomarkers for the cancer cells circulating in the body before the symptoms of the disease appears.

The availability of highly sensitive biosensors for early detection and diagnosis may provide the possibility of screening all male individuals at a much younger age than what is recommended at present for PSA. This could minimise the risk of late diagnosis of PCa and the related complications. In addition, biosensing methods could allow molecular profiling which would be of assistance to clinicians to identify novel subgroups of individuals with higher susceptibility to aggressive PCa development. Such high-risk subgroups may then be monitored more closely and frequently and at a much younger age than those currently undergoing routine PSA testing.

Whilst state-of-the-art in PCa screening relies on detection of PSA, these methods struggle to achieve detection limits of ng/ml of this biomarker. To enable detection of cancerous, circulating cells biomarkers before the symptoms of the disease appears new biosensors are needed: low levels of PCa biomarkers such as the prostate-specific membrane antigen (PSMA) are present in blood up to 5 years before diagnosis (Preston et al., 2019; Pang et al., 2020). Promising candidates as cellular biomarkers for earlier PCa detection and better diagnostic discrimination include up-/down-regulated membrane proteins in circulating PCa cells, (Damborský et al., 2016; Mansi et al., 2016) e.g., PSMA and/or gastrin-releasing peptide receptors (GRPRs) (Wright et al., 1996). These biomolecules are not yet routinely probed for in clinical settings (Perry et al., 2020).

To detect cancer early and personalised treatment, clinical testing stratifies the patient population e.g., by age and/or severity of disease. The successful early detection can be ensured through the accurate measurement/monitoring of new host biomarkers. The development of fast reliable methods for detecting PCa by electrochemical (Hofmarcher et al., 2020) or optical biosensing (Peña-Bahamonde et al., 2018), could address the unmet need of early diagnosis and monitoring non-invasively the response of PCa to treatment.

Imaging shows a significant role in the visualization and identification of tumour cells. It can provide essential information for making precise decisions for the treatment and accurate clinical management of a patient. There is a variety of imaging tests that can be used for different theragnostic applications of cancer. They can be used to predict whether a tumour is cancerous and if a biopsy is required. They can also recognised the stage of the cancer and the possibility of metastasis. Furthermore, they are readily used to monitor the progress of cancer after treatment and any chance of recurrence. Effective diagnostic imaging is based on the spatial resolution and contrast to noise ratio (CNR) of an image (Frangioni, 2008). Clinical imaging methods have become crucial techniques for detection and localisation of prostate cancer. Thanks to these methods, the stage and spreading of the cancer cells can be identified, and crucial inforamtion for the appropriate treatment can thus be delivered. Several improvements for each technique have recently been accomplished; however, the choice of imaging modality depends on the biological behaviour of the tumour (Hou et al., 2009; Sarkar and Das, 2016).

**FIGURE 1:**
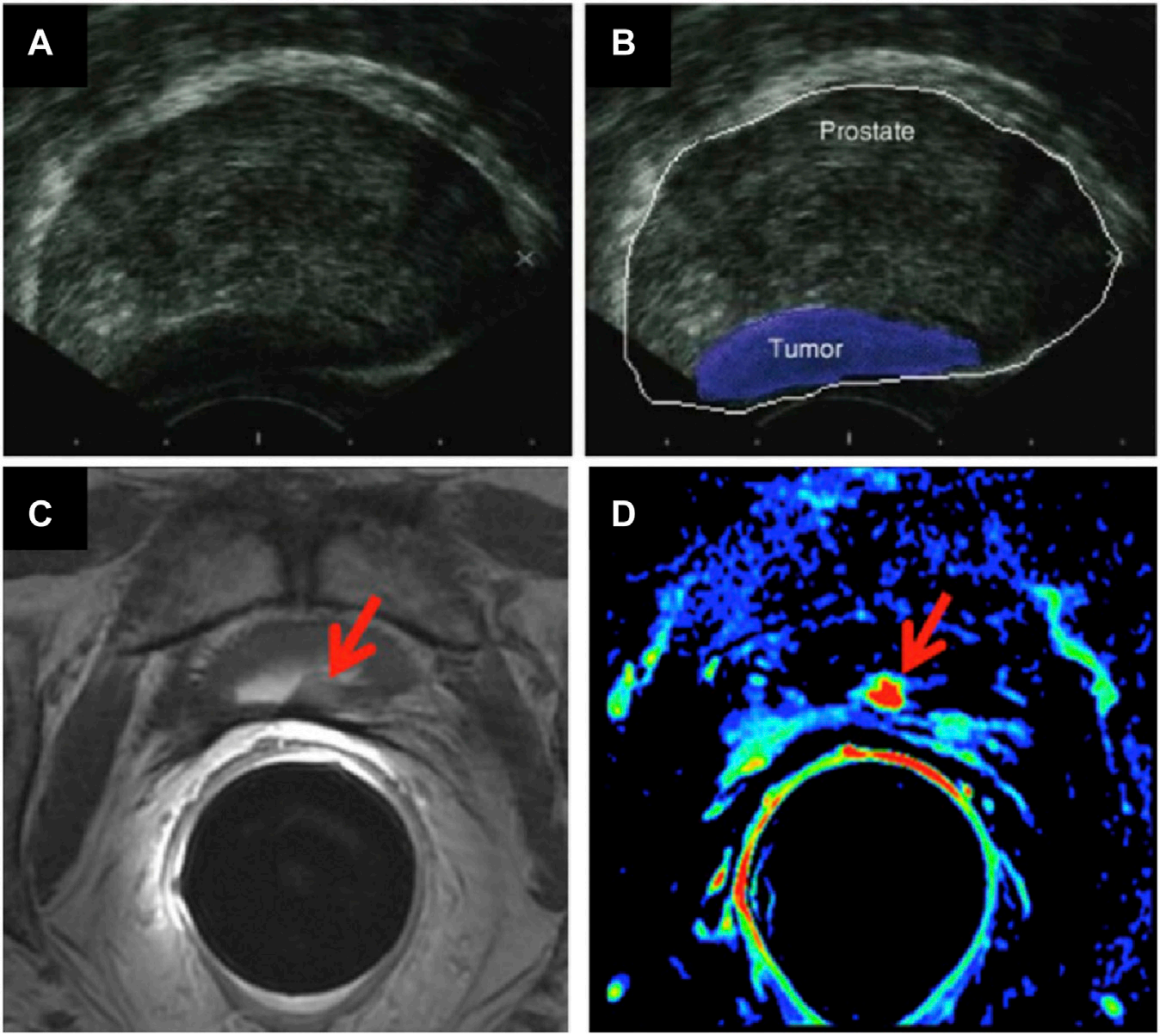
**(A,B)** TRUS images of a prostate affected by cancer (figure adapted from (Theodorescu and Ehdaie, 2009)); and MRI images of the prostate gland, showing cancerous regions (arrows): **(C)** Axial T2-weighted fast spin-echo image and **(D)** axial gradient-echo T1-weighted colour map image (figure adapted from (Maurer et al., 2016)).

The most common current PCa clinical diagnostic tests involve collection of tissue samples, or performing biopsies, which carry a risk of infection in some instances. There are two current methods of getting biospies: transrectal ultrasound guided (TRUS) biopsy, which comes with a link to serious infections, and a local anaesthetic transperineal (LATP) biopsy, recently approved by the National Institute for Health and Care Excellence (Nice), which is deemed to be safer in terms of side effects. It remains the case that Transrectal ultrasound imaging (TRUS) is the method of choice used when PCa is suspected (Pinto et al., 2011). The analysis consists of introducing an ultrasound probe in the patient’s rectum. This probe sends and receives sound waves through the rectal walls into the prostate which are elaborated by a computer into an image (Terris and Stamey, 1991) (Figure 1A). This technique determines the volume of the prostate and can help to evaluate if a cancerous tumour is involved.

**FIGURE 2:**
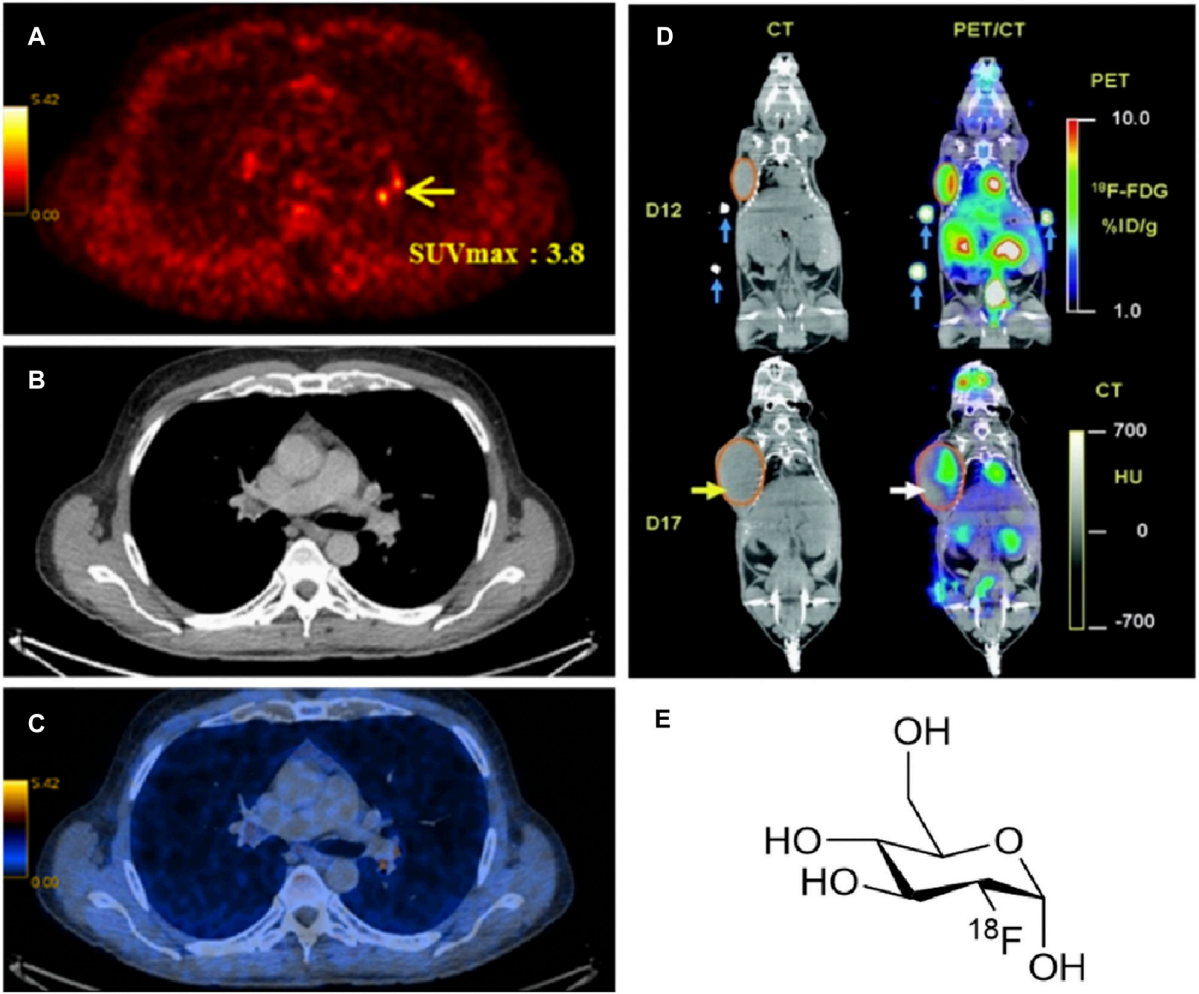
**(A)** PET image, **(B)** CT image and **(C)** PET/CT images of a male body with prostate cancer (Vali et al., 2015) (SUV: standardised uptake value (Kinahan and Fletcher, 2010) of ^18^F-fluorocholine). **(D)** Comparison between CT and PET/CT scans for the uptake of [^18^F]-FDG at day 12 and day 17. (Figure adapted from reference (Berger, 2003)), and **(E)** the structure of [^18^F]-FDG radiotracer.

The following imaging methods are minimally invasive and deemed to be precise and accurate to detect and localise cancerous tissues:

a) Magnetic resonance imaging (MRI) is based on nuclear magnetic resonance (NMR) (Rais-Bahrami et al., 2013). The involved physical process depends on adsorption and emission of energy by an atomic nucleus, placed in a magnetic field. In MRI, the nuclei of hydrogen atoms are used as probes for the detection of tissues because of natural abundance in fat and water. Varying parameters of the pulse sequence, different contrasts of the surrounding tissues can be obtained which are processed as images. This method is used to evaluate if the cancer is confined in the prostate or has metastasised in other parts of the body (de Rooij et al., 2014) (Figure 1B). Radio waves are being used to excite proton components of tumour cells to emit back radio waves. Different types of tumour cells can be differentiated as they emit different intensity of signals, depending on their size and composition (Scher et al., 2008). The rate at which proton nuclei spins returned to their original orientation is recorded to assess any abnormalities. Signal intensity is related to spin-lattice relaxation, T1 and on spin-spin relaxation, T2 of water protons of the tissue. Three -dimensional images can be obtained with high resolution. This technique is much more efficient for imaging soft tissues (Han et al., 2017). Detection of tumour can be improved by injection of an exogeneous contrast agent (Frangioni, 2008). Paramagnetic nanoparticles (NPs) can be used as T1 or T2 contrast agents, enabling the reduction of the relaxation rate of water protons. Most common T1 contrast agents are based on paramagnetic Gd^+3^ NPs where Iron Oxide NPs for example, are used as T2 contrast agents. The challenge of this method is to ensure that a specific tumour tissue has high affinity for one of the contrast agents used (Estelrich et al., 2015).

b) Single-Photon Emission Computed Tomography (SPECT), whereby long lived radioisotopes with half-life of many hours and days, are also used to perform more advanced PCa diagnostic scans. To enhance imaging signal, these radionuclides are able to bind to biomolecules like proteins in the form of a biomarker. The biomarker then drives to the tumour site, where it is concentration is being monitored using a gamma ray camera. When radioactive decay of the isotope occurs, it emits a gamma-ray (photon) of specific energy (Frangioni, 2008). Images can be taken, to highlight the location that the radioactive marker has been taken up in the body. Hence, it is easier to detect earlier the stage of the cancer and provide a better treatment. Radioactive decay of isotopes provides even more availability on radioactive elements to be used in imaging techniques. For instance, ^235^U can easily undergo neutron bombardment to split into its daughter isotopes, ^134^Sn and ^99^Mo *via* a fission reaction. The further decay of ^99^Mo to ^99m^Mo could introduce a significant diagnostic agent, this can enable the localisation of a tumour site by emitting γ-rays, a high energy radiation with a very low wavelength that can easily be removed from the human body (Paterson et al., 2014).

c) Positron-emission tomography (PET) and PET coupled with computed tomography (PET CT) are used to obtain information about the advanced structure and function of tissues *in vivo*. In PET, radiolabelled compounds are injected into the bloodstream of the patient. The radioactive compound presents half-lives enough long to be absorbed by the patient’s tissues and organs and afterwards record a scanned image of the relevant area. The radioisotope emits a positron through a positron emission decay (β^+^-decay) which travels in tissue for a short distance, typically 1 mm. During this path, the positron interacts with an electron (annihilation), producing a couple of photons (γ-photons) which moves in the opposite direction from the annihilation event. The γ-photons create a burst of light which is detected by a scintillator, converted in electrical signals and computed as an image (Figure 2).

PET radiotracers vary from ^11^C with 20.3 min half-life to ^64^Cu with 762.1 min half–life. Other available radioisotope is ^68^Ga with a half-life of 67.7 min (Evans et al., 2018). Imaging can be obtained by observing any biochemical alteration upon interaction of the tissue with the radiotracer (Scher et al., 2008). For instance, the metabolic rate of sugar with a radiolabelled tracer can be measured. Firstly, the patient is being injected with a radioactive tracer labelled with ^18^F or ^11^C. The radioactive decay of this tracer is being detected due to positron emission of the radioisotope. A positrons has the opposite charge of an electron (+1) and is being emitted from a proton-rich nucleus (Frangioni, 2008).

Radiolabelled tracers appear to be great imaging probes for detection of cancer. Firstly, such radioisotopes can be prepared in cyclotrons or by radionuclide generators. With the former being more expensive as one isotope is produced at a time, in comparison to the latter where long-lives parent isotopes can decay to short-lives daughter isotopes. These radioisotopes can then be easily separated by either ion-exchange chromatography or solvent extraction (Anderson and Welch, 1999). The challenge in deciding which radiotracer to use for which imaging method is focused on the capability of the tracer to target a specific site. The biodistribution, the clearance rate and specificity of binding of the radionuclide can affect the efficacy of the imaging probe. Besides, optimisation of the half-life of the radioisotope to be used could ensure efficient accumulation on a tumour site and consequently minimise the radiation dose towards the patient (Imlimthan et al., 2018).

A well-known radiolabelled tracer is 18-fluorodeoxyglucose ([^18^F]-FDG) which can be used to distinguish a healthy tissue from a cancerous tissue. This radioactive tracer showed to accumulate at the position of a tumour during its transfer by glucose proteins like glut-1 or glut-2. It can be easily diffused down the concentration gradient into the cell membrane of a tissue. Hence, PET/CT is effectual in detecting the location of a tumour and point easier the presence of cancer (Berger, 2003). This statement is confirmed using an animal model similar to that shown in Figure 2D. During the experiment, a mouse has received a small dose of the PET radiopharmaceutical [^18^F]-FDG (Figure 2E). The uptake of the radiotracer was recorded using CT scan and conjugate PET/CT image that shown a more precise location and size of the tumour.

Compared to other types of cancers, the use of [^18^F]-FDG in PCa could result to a limitation, due to the very low metabolic rate and low slight uptake of the tumour cells. Under these circumstances, PET tracers like [^11^C]-Choline could be better replacements (Kai, 2010; Tegler et al., 2012). Choline is an essential component of the cell membrane of a tissue. Upon injection in the body, it can easily be labelled with ^11^C or ^18^F due to their short half-life. The spread of cancer can be directed, due to the extent of choline uptake that provides evidence of tumour cell proliferation (growth) (Bouchelouche et al., 2010b). Generally, ^18^F radioisotope is used in PET tracers due to its high half-time of 109.8 min in the body. Monoclonal antibodies (mAbs) are also be used for targeted imaging, but the requirement of longer-lived radioisotopes like ^124^I and ^86^Y is high. They can provide a slower clearance rate from the body due to their large size (Imlimthan et al., 2018). Overall, the resolution and sensitivity for each technique vary. For comparison, SPECT, PET and CT are ionizing radiations that can cause mutilation to healthy tissue where MRI is a non-ionizing technique but relatively an expensive method (Fass, 2008). Table 1 summarises several advantages and disadvantages of the imaging techniques discussed.

**TABLE 1:** Advantages and disadvantages of imaging techniques.

Type of imaging technique	Advantages	Disadvantages
CT	Fast and painless process	Costly and X-ray exposure
SPECT/PET	Fast detection and painless process	γ-ray exposure
MRI	Non-ionising radiation	Costly, noisy, reports of patients claustrophobia

In the early 2000s, PET and CT had become important medical techniques thanks to their multimodal application (Townsend, 2008). In addition, accurate and detailed images are obtained in a single scan because of the combination of information about body’s anatomy, deriving from CT scans, and metabolic functions, obtained from PET scans (Figure 2C). For CT imaging arrow beams of X-rays are shot by the rotating tube around the patient situated on the scanner and 2D image slice of the organ or tissue are generated by each scan. The collected image slices are elaborated by a computer which generates a 3D image of the scanned patient’s body part (Figure 2B). Experimental studies showed that a combination of the techniques described above with Computed Tomography (CT), can allow an even better analysis for the incidence of a tumour tissue and if it is present, its location and stage could be determined. This method was found to be efficient as a diagnostic tool as it can allow the detection of a larger tumour in comparison to MRI which provide information only for a soft tissue (Berger, 2003; Jadvar, 2013). The CT technique can produce cross-sectional images using X-rays. These images permit the morphology and structure of a tumour tissue to be observed. Therefore, a hybrid combination with CT will be very beneficial as it allows imaging to be carried out in a single process with high precision and validity of the tumour location.

Recently, a hybrid combination of MRI/CT showed a more improved process of detection and prognosis of cancer (Bouchelouche et al., 2010b). The following two methods are based on radiotracer imaging probes. The half-life and the decay pathway of the radiometal to be used should be considered for each case. Other significant factors for deciding which radionuclide to use is its cost and availability (Anderson and Welch, 1999). MRI and PET/CT have become important techniques for detecting, localising and evaluating both the stage and position in the human body of prostate cancer. Despite their improvements in accuracy, sensitivity, and precision, they are considered expensive for many patients and accessible only for few medical structures to perform an early-stage diagnosis for prostate cancer.

Overall, a variety of imaging techniques have progressed to the clinic and are accessible for use in all stages of cancer. They constitute a library of tools able to predict and locate earlier and easier whether a tumour is present in the human body. The resulting images can offer a detailed analysis of the morphology and anatomy of a tumour cell and therefore could contribute to the decision-making for the appropriate treatment. Many factors should be considered for the correct choice of an imaging technique, with the most clinicians concentrating on the health and safety issues (Jadvar, 2013). Nowadays, imaging is used as a screening technique when a patient has already shown some symptoms. Many fields, including biotechnology, pharmaceutical industries and nanotechnology target to offer an early pre-symptomatic detection of cancer to minimize death rate due to cancer (Fass, 2008). This objective proves to have a very high potential that can be expanded in research, offering more efficient targeted imaging using nanoparticles as contrast agents (*vide infra*).

**FIGURE 3:**
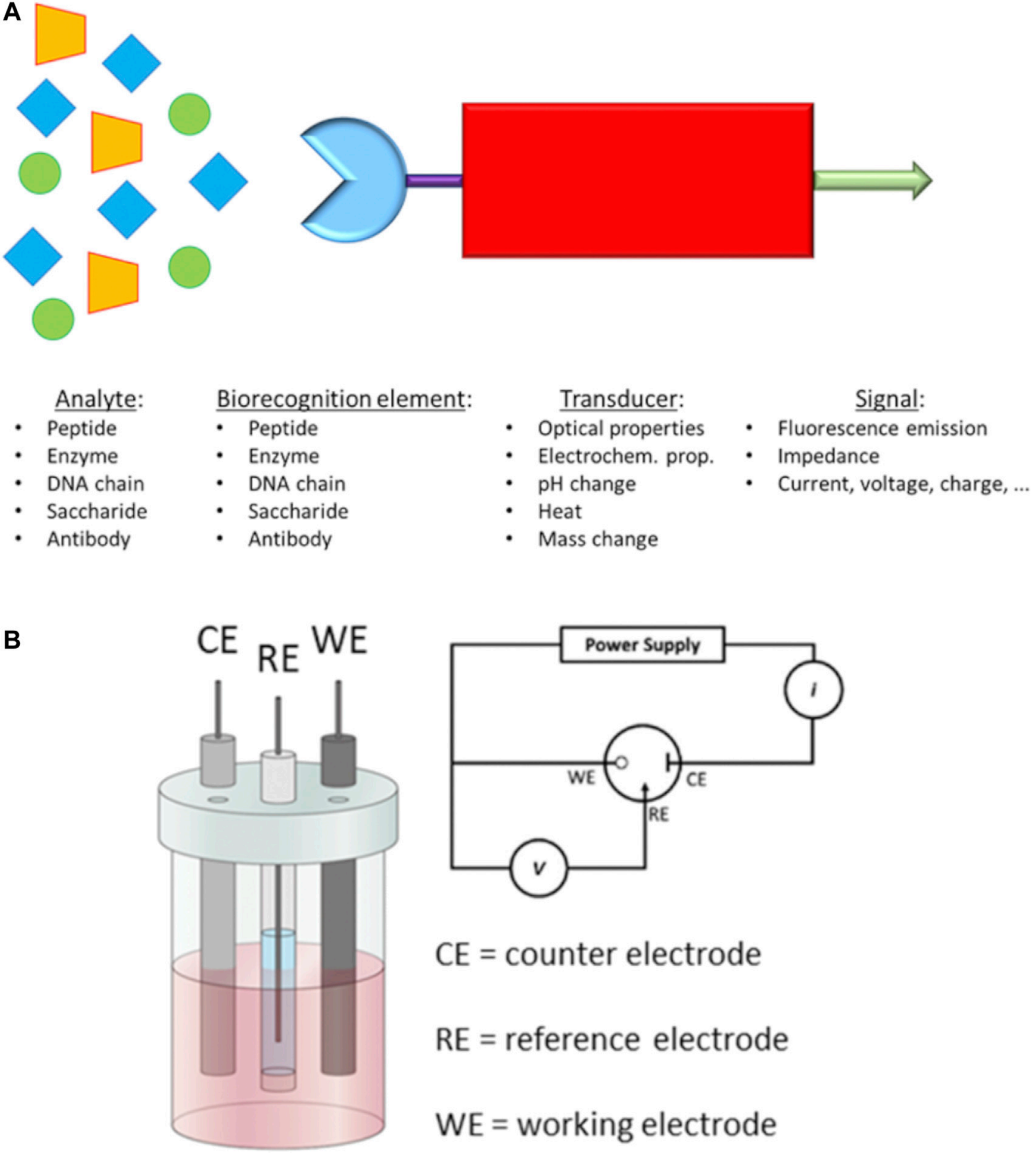
**(A)** Schematic representation of a biosensor and **(B)** Electrochemical 3-electrodes cell and its relative electric circuit (adapted from Raccichini et al. (2019) and Fischer et al. (2009)).

d) Point-of-care tests (POCTs) have become widely used as early-stage diagnostic methods to detect specific biomarkers on the occurrence of a disease. POCTs are affordable, quick and ready-to-use solutions which do not need a specialised medical centre and/or staff. For example, the PSA test is one of the most common POCTs used by doctors to assess the occurrence of PCa in the early stage. Although PSA test has helped to decrease the mortality for PCa (Barry, 2001), it can lack accuracy and specificity for prostate cancer. In fact, other non-cancerous diseases of the prostate, such as benign prostatic hyperplasia (BPH) (Verhamme et al., 2002), can also increase the level of released PSA in the blood, giving a false-positive result for prostate cancer. Furthermore, PSA tests usually are performed in dedicated laboratories which require large and automated analysers, sample transportation, long waiting time and costs for administration and medical staff. The burden of research field on PCa is to recognise selectively and in low concentration some biomarkers produced in the early stage by cancer cells. Generally, these tests are based on biosensors which are capable to interact with a specific biomarker produced during a disease. The most common parts in a biosensor of this kind are I) a biorecognition element (i.e., antibodies, saccharides or peptide fragments) which selectively recognises and binds the analyte; II) a transducing component converts the interaction of biomolecules in a quantifiable signal; III) a readout system is used to read the results (Figure 3A).

The transduced signals obtained by the biosensor can be an electrochemical signal or a variation of fluorescence intensity, optical biosensor (Devillers et al., 2017; Jayanthi et al., 2017; Mittal et al., 2017; Pan et al., 2017; Yavas et al., 2017). An important requirement for an optical biosensor is the presence of a fluorescent moiety in which gives a variation of fluorescence intensity once a binding event occurs with the analyte. On the other hand, electrochemical biosensors employ electrical property changes (for instance, voltage, current or impedance) to detect the binding event with the targeting analyte. Such produced signal by these types of biosensors is proportional to the concentration (Pohanka and Skládal, 2008; Hammond et al., 2016) of the targeting analytes. These concentration-dependent properties have been challenging the research field of biosensor design and construction to reach sensitivity and specificity at low concentration of the analyte. In this regards, different research groups have reported novel molecular biosensors, either fluorescent or electrochemical (Zhu Y. et al., 2016; Topkaya et al., 2016), able to detect specific biomarkers related to a disease, e.g., the PSA level for prostate cancer (Jolly et al., 2015). In addition, these biosensors can be embedded in inexpensive, portable and ready-to-use devices able to perform point-of-care tests at any time without specialised medica staff (Leng et al., 2010).

In the past decades, novel electrochemical biosensors, which use electrical impedance spectroscopy (EIS) as a method for prostate cancer detection, have been increasingly exploited in the research field. EIS can use label-free detection which consists of direct, sensitive and real-time sensing of the binding event between the biorecognition element and analyte (Mehrabani et al., 2014) measured in the form of either capacitance or resistance changes (Arya and Bhansali, 2012). Such electrical properties are related, through mathematical equations, to impedance. EIS measurements are based on the detection of the impedance in an electrochemical cell which works in the alternate current regime. The electrochemical cell employed for the EIS measurements consists of a 3-electrode cell with a counter electrode (CE), a reference electrode (RE) and a working electrode (WE) (Lee et al., 2017) (Figure 3B).

The function of the CE is to close the circuit and maintains the reference potential ideally constant; on the other hand, the RE presents a known potential and is used to obtain the potential difference between the WE and RE. The WE presents a biorecognition element on the electrode surface, generally gold, which binds the target molecules in the solution. A potentiostat is used to apply a desired alternate potential between WE and RE. In the last decade, this setup has been increasingly revised in order to build miniaturised electronic devices to perform point-of-care diagnosis for the detection of PCa biomarkers (Chornokur et al., 2011; Chiriacò et al., 2013; Pihíková et al., 2016). One of the recent miniaturised EIS-based biosensors (Ibau et al., 2019) has shown excellent responses for the detection of PSA level in human serum, comparable to the clinical threshold of 4 ng ml^−1^. Such biosensor can represent an example for the development of novel devices for early-stage detection of PCa biomarkers thanks to the small size, low-cost, sensitivity, reproducibility and reliability (Ibau et al., 2019).

### 2.1 Synthetic Scaffolds for Biosensing Applications in Living Cells

As previously mentioned, the construction of a biosensor requires a transducer which links the biorecognition element and generates the physical property to be converted in a signal, for example, electrochemical (Ronkainen et al., 2010) or optical (Damborský et al., 2016) responses.

Several organic molecules have been discussed as synthetic scaffolds of interest as tool-box componets for the PCa biosensing probes. Amongst these, the class of molecules denoted naphthalenediimides (as functional derivatived of 1,4,5,8-naphthalenetetracarboxylic diimides, NDIs) became of interest over the 30 years. These compunds have been widely explored in different research fields such as electronics, material science, biology and medicinal chemistry (Al Kobaisi et al., 2016) due to their potential to link different moieties on the construction of molecular devices as well as contribute to sensors design and development. NDIs are derived from naphthalene dianhydride (NDA) and an amine and represent the smallest homologue of rylenediimides (RDIs). The first application of NDI derivatives was as synthetic pigments and dyes (Thalacker et al., 2006). NDIs present an electron-deficient aromatic π system which can be extended to show a wide range of absorption and fluorescence emission wavelengths (Thalacker et al., 2006). Simple NDI derivatives present absorption maxima at around 350 nm which can reach a range from 500 to 760 nm (Thalacker et al., 2006) with proper derivatisation. In addition, their main characteristic is high fluorescence quantum yield and fluorescence emissions in a range between 650 and 780 nm (Thalacker et al., 2006).

The ease of functionalisation of NDIs with aminoacids and peptides has been increasingly exploited for modulating their optical characteristics, suitable for the optical imaging applications, and recent work has shown that they cell penetrate living prostate cancer cells (Hu et al., 2015). The major contribution of the characteristics of NDIs is due to modifications through the derivatisation of the aromatic core which can vary both electrochemical and optical properties. For example, aryl derivatisation in position 2, 3, 6 and 7 can lead to species with a quantum yield close to 1 in solvents of various nature (*i.e.,* aliphatic, aromatic, chlorinated and dipolar) (Al Kobaisi et al., 2016). On the other hand, the derivatisation in the imide bonds results in little effects on the absorption and fluorescence spectra of NDIs. The planar aromatic system of NDIs is responsible for aromatic π-stacking and Van der Waals interactions with other aromatic species. In fact, most aromatic rings present a quadrupole moment created by their electron density with a partial negative charge above and below the face and a partial positive charge around the periphery (Prentice et al., 2017).

In different fields, the interactions created by both the planar aromatic core and substituents on the NDIs have generated different devices such as organic solar cells (Earmme et al., 2013; Hwang et al., 2015), field-effect transistors (Guo et al., 2012; Yuan et al., 2016), sensors (Cox et al., 2012; Zong et al., 2016) and catalysts (Senkovskyy et al., 2011; Martinez et al., 2017) alongside their use as imaging probes (Hu et al., 2012, Hu et al., 2015; Tyson et al., 2016).

**FIGURE 4:**
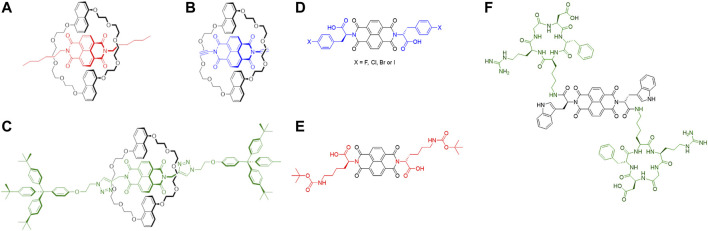
**(A–C)** Examples of NDI-based rotaxanes. a) *Pseudo*rotaxane with alkyl chains on the NDI core as UV-visible molecular switch (Pascu et al., 2007); **(B)** NDI-based *psuedo*rotaxane intermediate for the formation of [2]-catenane (Hamilton et al., 1998); **(C)** NDI-based [2]rotaxane as electrochemical induced shuttle (Jacquot de Rouville et al., 2012) **(D–F)** Some examples of amino acid/peptide-tagged NDIs. **(A)** NDI core tagged with halo- *l* -phenylalanine (Hu et al., 2012; Tyson et al., 2016) (in blue); **(B)** NDI core derivatised with *l* -lysine, protected with *tert*-butoxy carbonyl protecting group (Răsădean et al., 2017) (in red); **(C)** NDI core tagged with cyclo (RGDfK) peptide (Hu et al., 2015) (in green).

Mechanically interlocked molecules (MIMs) (Griffiths and Stoddart, 2008; Bruns et al., 2014) including those based on rotaxanes (Figure 9) have been explored in a wide range of applications such as catalysts (Du et al., 2017), chemical sensors (Brown et al., 2017), polymers (Ahamed et al., 2017), molecular switches (Zhu N. et al., 2016) and motors (Wilson et al., 2016). In addition, these types of self-organised compounds have also found application in the biological field as a system for drug delivery (Shi et al., 2015), target bacterial protein (Vincent et al., 2016) and release of bioactive peptides (Fernandes et al., 2009). Recently, the optical and fluorescent properties of rotaxanes have been exploited for the design and synthesis of imaging agents such as MRI agent (Fredy et al., 2017), mitochondrial stainer (Yu et al., 2016) and fluorescent dye (Lee J.-J. et al., 2013). In this context, Naphthalenediimides have increasingly become interesting components for the design and formation of rotaxanes and pseudo-rotaxanes (Figures 4A–C). (Pan et al., 2011; Slater et al., 2011; Bruns et al., 2014).

NDI-based conjugates increasingly investigated for their potential to act as imaging agents or as building blocks for more complex supramolecular architectures with sensing and imaging applications. The introduction of amino acids on the NDI core have been investigated for water solubility and application in biological media. Such amino acids-tagged NDIs can be obtained by microwave-assisted synthesis which has become a fast, reliable, efficient and high yield method to symmetrise and asymmetrise the NDI core (Pengo et al., 2006; Tambara et al., 2011). In the last decades, different amino acid-tagged NDIs have been studied in aqueous solutions to investigate their aggregation and behaviour in such media (Avinash and Govindaraju, 2012; Nandre et al., 2013; Hsu et al., 2015). These species tend to aggregate with themselves and other aromatic compounds thanks to the presence of the extended electron-deficient π-system on the NDI-core. This characteristic has been explored with electron-rich aromatic materials such as carbon nanotubes (Hu et al., 2012) and graphene congeners (Hu et al., 2012) to create supramolecular aggregates for novel bioimaging probes (Figure 4D). In these works, the fluorescence and aggregation properties of such amino acids-tagged NDIs have been explored in addition to their biocompatibility in living cells. The fluorescence properties in living cells of such compounds have been extended to the NDI core which links peptide moieties on its sides (Figure 4F). Such peptide, cyclo (RGDfK), has shown an affinity to α_v_β_3_ integrins which are overexpressed during the angiogenesis of some kind of cancers, such as prostate (Hu et al., 2015). This peptide-tagged NDI has shown uptake and distribution in living prostate cancer cells with good integrity as imaging probes for the detection of cancer cells *via* fluorescence spectroscopy.

The biocompatibility, water solubility and capacity to aggregate with electron-rich species of amino acid-functionalised NDIs have also inspired some works to bind G-quadruplexes as potential anticancer therapies (Figure 4) (Răsădean et al., 2017) These species have shown recognition and affinity to such G-quadruplexes which are driven thanks to aromatic stacking and hydrophobic interactions to create supramolecular aggregates.

## 3 Molecular Imaging Approaches to Drug Discovery Processes

Imaging can have a broad depth of applications from lab towards clinical practise. It is established to be a great tool that provides evidence in the form of scans and images to predict the efficiency of a potential new drug. Designing a new drug and introducing it to patients can be a very costly and time-consuming multi-staged process. Collaborations of pharmaceutical industries and research organisations, attempt to critically decide which drug to spent time investigating and which one to stop emerging based on their potential to offer better treatment and medication (Murphy, 2010).

Firstly, a clear understanding of the molecular mechanism focused on a pathology is required to allow a particular target selection. Validation of a potential drug target involves imaging methodologies at a molecular and structural level. Once a target is being recognised a compound with high selective affinity for the target is essential. The target affords a signal and subsequently a precise prediction of the efficacy, safety and biocompatibility of a candidate drug. During this lead optimisation step, animal testing was found to be fundamental for translating theory into clinical practise for a human disease. The next stage that the potential drug has to be authorised, is the clinical advance trial (Rudin, 2005). Imaging enriches clinical trials, as data analysis will give evidence of the extent to which a tumour has been minimised in size. For example, the use of biomarkers can show the response of the drug on tumour site and the progression of the disease. This approach drives towards a faster prognostic therapy. Before the clinical trial for a candidate drug a pre-clinical phase is required to demonstrate how a drug behaves in the body and recognise any potential safety hazards towards patients (Takayanagi et al., 2004). Recent research trials encounter many limitations such as the availability of a standardisation factor. Thus, pharmaceutical industry focus on the development of more efficient targeted imaging techniques to provide a more accurate prediction of therapy (Yankeelov et al., 2016). We will first review the use of some molecules with excellent properties as imaging agents and then focus on nanotechnology and the great potential it offers to solve current problems in the field.

### 3.1 Small-Size Peptides as Targeting Agents

In the late 1990s, monoclonal antibody-based therapy was established and used to treat patients with solid tumours (Aina et al., 2007). This strategy is considered one of the most successful and important to target specific receptors which are overexpressed, mutated or selectively expressed by human cancer cells (Scott et al., 2012). Most of the approved monoclonal antibody therapeutics can inhibit tumour growth, target specific antibodies and deliver radionucleotides or toxins to cancer cells (Aina et al., 2007).

**FIGURE 5:**
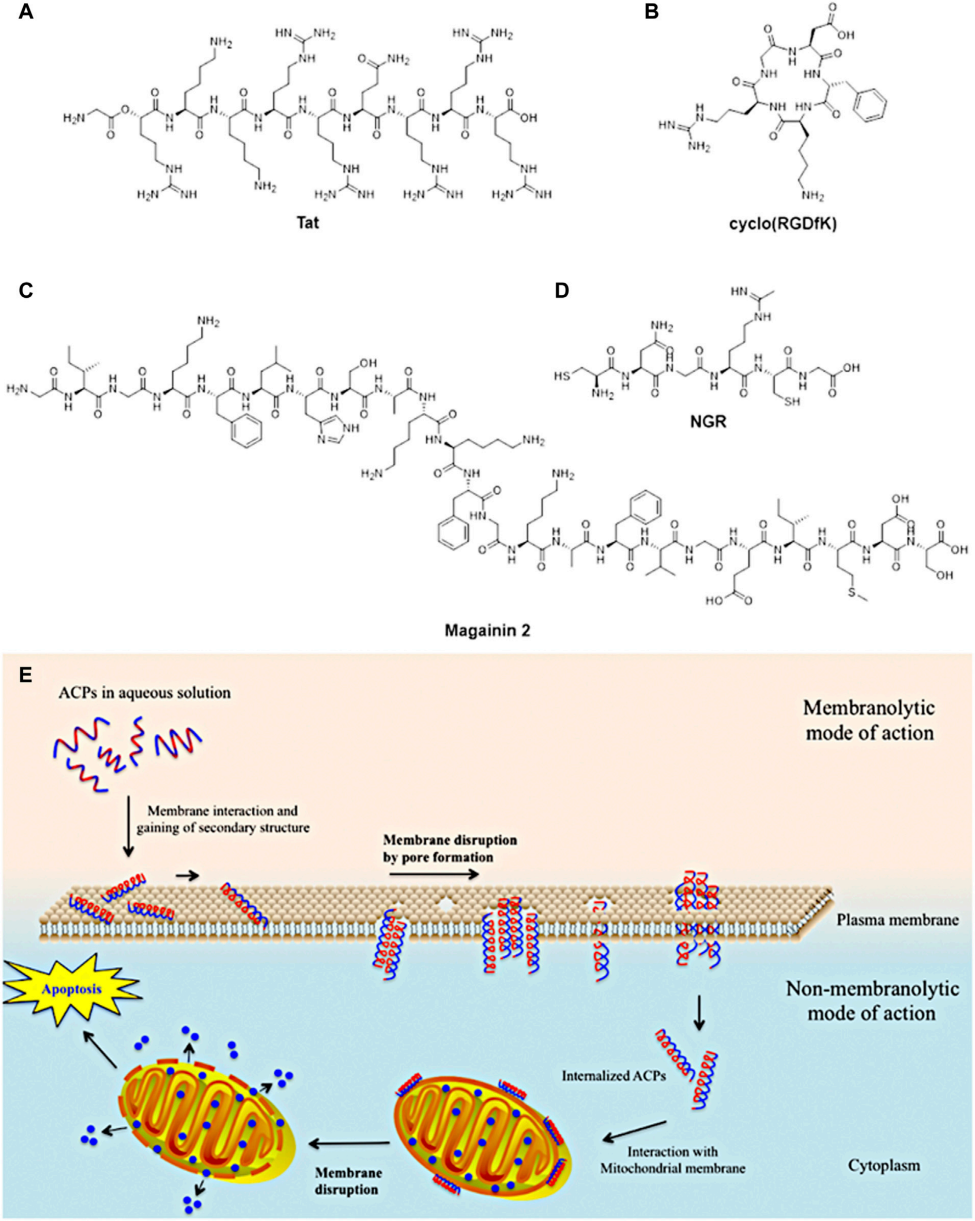
Examples of small-sized peptides in pre-clinical and clinical trials of therapeutic agents. **(A)**Tat peptide for HIV therapeutic use; **(B)** cyclo (RGDfK) peptide for anticancer therapy such as breast, ovarian and prostate; **(C)** magainin 2 peptide for bladder anticancer agent and antimicrobial for diabetic ulcers; **(D)** NGR anticancer therapy such as ovarian, lung and colon (figure adapted from Boohaker et al. (2012)). **(E)** Proposed mechanism of action of anticancer peptides into cancerous cells (figure adapted from Tyagi et al. (2015).

Despite such characteristics, monoclonal antibodies have been showing some limitations such as poor diffusion into tumoral cell membranes due to their large sizes and non-specific uptake by liver or bone marrow (Zhang et al., 2012) of radionucleotides, cytotoxic drugs or toxins (Aina et al., 2007). Considering these restrictions of monoclonal antibodies, cancer research has moved to design and synthesise small-sized peptides (*circa* 3,000 Da (Aina et al., 2007) or below 50 amino acids (Marqus et al., 2017)) which can mimic a portion of antibodies or being complementary to receptors on the surface of cancerous cells. These peptides have shown many advantages such as ease to synthesis (Boohaker et al., 2012; Thundimadathil, 2012), better penetrability into tumoral tissues due to their small size (Lau and Dunn, 2018), capacity to target specific receptors on cancer cells surface and have lower toxicity to liver and bone marrow (Thundimadathil, 2012; Zhang et al., 2012). These characteristics have been increasingly exploited for different applications for biomedical purposed such as anticancer and antimicrobial agents and tumour targeting (Figure 5).

Another application of small-peptides is the use as carries for drug delivery of bioactive portion into the cells thanks to the affinity and recognition of receptors on cell surface (Jitendra et al., 2011; Nasrollahi et al., 2012; Svensen et al., 2012; Ruoslahti, 2017). Once the peptide is recognised by the cell receptors, it can go into the cells and releases the active drug which can kill the cancerous cell.

Moreover, small-sized peptides have been studied as potential alternatives to synthetic drugs to anticancer treatment. Anticancer peptides (ACPs) have shown some advantages such as low toxicity for healthy cells and tissues, efficacy, selectivity and specificity for cancerous cells (Chen W. et al., 2016). Different anticancer peptides have been designed to interact with the cellular membrane, able to disrupt it and penetrate inside the cell (Gaspar et al., 2013). This peptide can interfere with necrotic and/or apoptotic mechanisms of the cancerous cells, leading to the cell death (Gaspar et al., 2013). Despite such characteristics, these peptides require an accurate and specific design to perform those processes.

Another class of anticancer peptides has been developed to target specific markers placed on the cellular membrane which can be expressed or overexpressed by cancerous cells (Marqus et al., 2017). Such targeting-peptides present portion of the receptors of cancer cells which can be selectively recognised and show high affinity to receptors in only cancerous cells. These criteria in addition to retention in the target, rapid clearance from healthy tissues or cells and high stability *in vivo* (Lee S. et al., 2010) have made targeting-peptides ideal candidates to design and synthesise imaging probes for clinical use.

In recent years, targeting-peptides have been increasingly combined with different fluorescent scaffolds such as nanoparticles (Steinmetz et al., 2011), fluorophores (Luo et al., 2011) and ligands for metals (Fani et al., 2012; Gaertner et al., 2012) to be detected through radionuclide-based and/or fluorescence imaging. The advantages of peptides anchored to such scaffolds have been inspired researchers to find novel targeting-imaging probes for cancer cells detection. In particular, multimodal imaging (*e.g.,* PET/MRI and PET/CT) have become an important method to cancer detection thanks to the advantage to administrate a single contrast agent for different imaging modalities and signal consistency at the target region without a difference in biodistribution which could occur using two different contrast agents (Lee J. et al., 2013).

Several multimodal peptide-based imaging probes (Kinsella et al., 2011; Sun et al., 2011; Lee J. et al., 2013; Key et al., 2016) have been reported in the last decades able to work in multimodal imaging domain for both *in vitro* and *in vivo* studies. For instance, ^64^Cu has been the most common radioisotopes, exploited in PET imaging for years, thanks to its sufficiently long half-life and forms stable complexes with different ligands. Ma and Donnelly (2011)_64_Cu with t ½ of 12.7 h and Eav = 278 keV) is both a beta+ and a beta- emitter which renders it suitable for both imaging and radiotherapy. The use of this metal radioisotope has most promising for successful imaging and therapy applications when the judicious choice of the bifunctional chelator enabled the facile conjugation to the targeting peptide or protein and radiolabeling conditions showed biocompatibility. Progress in this domain has been hampered by the necessity to use elevated temperatures over prolonged periods for efficient labelling of most common chelators such as DOTA or TETA. A less challenging chelating system reliant on N2S2 motif which was developed to show fast labeling kinetics at room temperature and at near-neutral pH. Additionally to the high radioincorpartion yields the use of functional CuATSM as a synthetic scaffold advantaged the labeling of sensitive peptide fragments unable to withstand elevated temperatures and extreme pH ranges (Hueting et al., 2010). Such characteristics have been combined with various peptides to design and synthesise PET imaging probes able to target specific receptors such as gastrin-releasing peptide receptors and integrins. Ma and Donnelly (2011) Significantly, a considerable number of preclinical investigations into the ^64^Cu labelling of peptides of relevance for prostate cancer cells and tumours targeting have been reported, including the development of Cu-copper sulfide nanoparticles and bombesin functionalised for targeted imaging of orthotopic prostate cancer (Cai et al., 2018).

Despite the advantages due to the use of peptides which make these imaging probes safer, the risk of radiations in the human body can persist. In addition, MRI and PET/CT can still lack specificity and sensitivity and moreover be invasive and expensive to patients. On the other hand, fluorescence imaging methods have been considered promising alternatives thanks to some advantages such as non-invasiveness, real-time, high resolution and low-cost (Luo et al., 2011). In addition, the range of wavelength of the near-infrared (NIR) (700–1,000 nm) has increasingly become fundamental in fluorescence imaging due to low absorption and autofluorescence of cells and/or tissues, deep penetration in tissues, non-invasiveness and sensitivity in image (Luo et al., 2011). The ability of small-sized peptides to target specifically cancerous cells, the tune-able fluorescence property of the scaffolds where such peptides are attached and use NIR wavelength range have widely increased the number of published works on imaging probes for cancer detection.

### 3.2 Bombesin-Related Peptides as Potential Targeting Groups for the Detection of PCa

Gastrin-releasing peptide receptors (GRPR) have been discovered in a variety of solid tumours such as breast, colon lung and prostate. In prostate cancer, their expression rates are in the range between 63–100% (Beer et al., 2012) and moreover, they upregulate promoters of angiogenesis, essential for the metastasis of the cancerous cells (Elshafae et al., 2016). In recent years, GRPRs have increasingly received a lot of interest for being targeted to treat and image prostate cancer (Ciaffaglione et al., 2021). These receptors have shown a high affinity to two natural peptides: GRP consists of 27 amino acids, found in mammals, which is homologous to the amphibian bombesin (BBN) peptide from the species *Bombina* (Anastasi et al., 1971; Spindel, 1986; Lee et al., 1994), a 14 amino acids chain (Schroeder et al., 2011; Beer et al., 2012). Thanks to their high affinity to the GRPR, such peptides have become important targeting compounds for prostate cancer cells. In particular, bombesin shares the same amino acid sequence (Gln-Trp-Ala-Val-Gly-His-Leu-Met-NH_2_) of the human GRP which has been found responsible for the high affinity to the receptors (Paterson et al., 2010). Among small-sized peptides, the fragments 7–13 and 7–14 of bombesin peptide have become important targeting species for prostate cancer and investigated in the context of imaging and therapeutic targets. Peptide fragments of this family have received particular interest in the biological research field because of the hypothesis that mammals present bombesin receptors which are abnormally expressed and/or overexpressed in some malignancies (Sancho et al., 2011). Mammalian bombesin receptors are classified in 3 subtypes: neuromedin B (NMB), gastrin-releasing peptide (GRP) and bombesin receptor subtype 3 (BB3) (Pooja et al., 2019). Such subtypes of receptors are involved in specific physiological processes such as immune defence, thyroid and adrenocortical function, deglutition, weight regulation and cognition for NMB; on the other hand, GRP releases gastrointestinal hormones, secretes pancreatic and gastric fluids and smooths muscle contraction (Elshafae et al., 2016).

The affinity and binding of one such sequence, called BBN [7–14], has been recently exploited in different works to design and synthesise targeting therapeutic and/or imaging agents for prostate cancer. In addition, the fragment 7–13 of the bombesin peptide has shown a similar affinity to the GRPR. In these regards, different fluorescent or radiolabelled imaging probes incorporating bombesin fragments have recently been reported–these rely on the affinity of either the fragments [7–13] or [7–14] of the bombesin peptide to the GRPR to achieve optical imaging, combined with SPECT and radio-therapy (Agorastos et al., 2007), or MRI (Jafari et al., 2015; Koo et al., 2015) and PET/CT (Schroeder et al., 2011; Inkster et al., 2013) imaging *in vitro* and/or *in vivo*.

**FIGURE 6:**
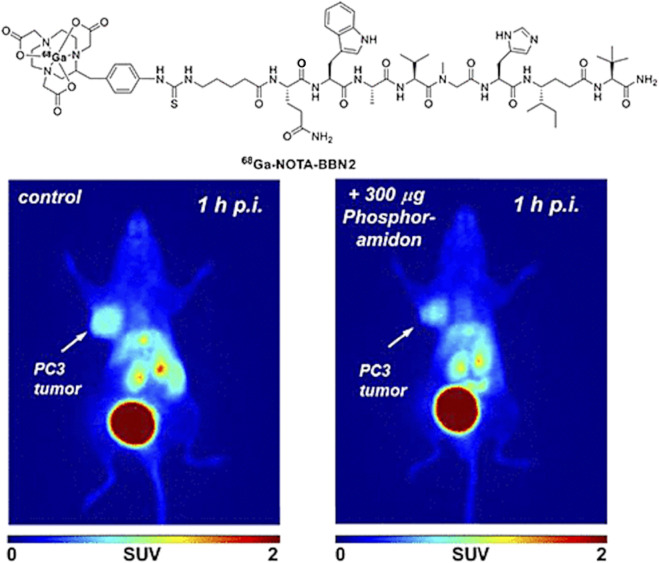
PET images obtained using [^68^Ga]-NOTA-BBN2 in PC3 (left) or LNCaP (right) tumor-bearing mice xenografts additionally to the considerable uptake in the gallbladder and the bladder. Reproduced from reference (Richter et al., 2016).

Some probe development as well as preclinical investigations involving ‘cold’ (Liolios et al., 2012) or radiolabeled ^64^Cu-bombesin conjugates have also been reported (Hueting at al, 2010), including assays *in vitro* and *in vivo* for radiopharmacological evaluation (Bergmann et al., 2013) Moreover, combined radiolabelled targeting-peptide compounds have also been studied as therapeutic agents which can interfere with cellular growth in prostate cancer (Cui et al., 2013; Morgat et al., 2014; Moreno et al., 2016). Several reports focused on derivatives of the full 14-mer peptide, typically functionalised at the lysine residue, to shorter fragments typically the [7–14] and [7–13] fragments which are N-terminally functionalised and fragments such as Lys^3^-BBN and BBN [7–13] along with [^125^I]-ITyr^4^BBN have been reported. The *in vivo* molecular imaging results obtained with such radiolabeled bombesin derivatives are highly dependent on the nature of tumours investigated *in vitro* or *in vivo* in preclinical studies. In typical PCa cell lines such as PC-3 (bone metastasis of a grade IV PCa) it has been shown that there are severely upregulated GRPR, other PCa cell lines, whereas the LNCaP cell line (lymph node metastasis of a prostate adenocarcinoma) express the GRPR to a lower level. Figure 6 shows the PET images obtained using a [^68^Ga]-NOTA-BBN2 derivative in tumor xenograft models and improved metabolic stability (Richter et al., 2016).

In terms of in-patients imaging the targeting of the Gastrin-releasing peptide (GRP) receptor has been investigated by scintigraphy from the hypothesis that this procedure could allow prediction of response to GRP receptor-targeted treatment options, early non-invasive diagnosis and *in vivo* prognostic stratification of GRP receptor-positive tumours (Van de Wiele et al., 2000). This publication from 2000 reports a pilot study approved by the Ethical Board of the University Hospital Ghent, assessing the safety, imaging characteristics and efficacy for tumour detection of the GRP analogue ^99m^Tc-RP527 developed for GRP-R scintigraphy. The report discusses the imaging characteristics and efficacy for tumour detection of technetium-99 m RP527, a ^99m^Tc chelated targeting peptide derived from bombesin, which was found to bind GRP receptors with high affinity. In male patients (n, number of patients = 4, mean age 56.4 years) suffering from metastasised prostate, data presented suggest that ^99m^Tc-RP527 results in specific tumour localisation and exhibits good imaging characteristics with a good T/N ratio that may be further enhanced by single photon emission tomography (SPECT), whereby ^99m^Tc-RP527 showed specific uptake in one of four prostate carcinomas. Whilst this report indicated the feasibility study, or pilot, nature of the investigations, it highlightes the fact that *in vivo* GRP-R scintigraphy could allow prediction of response for GRP-R targeted treatment possibilities and advance the prognoses for early non-invasive tumour diagnosis (Van de Wiele et al., 2000).

### 3.3 Radiolabelled Biomarkers for the Imaging of PCa

**FIGURE 7:**
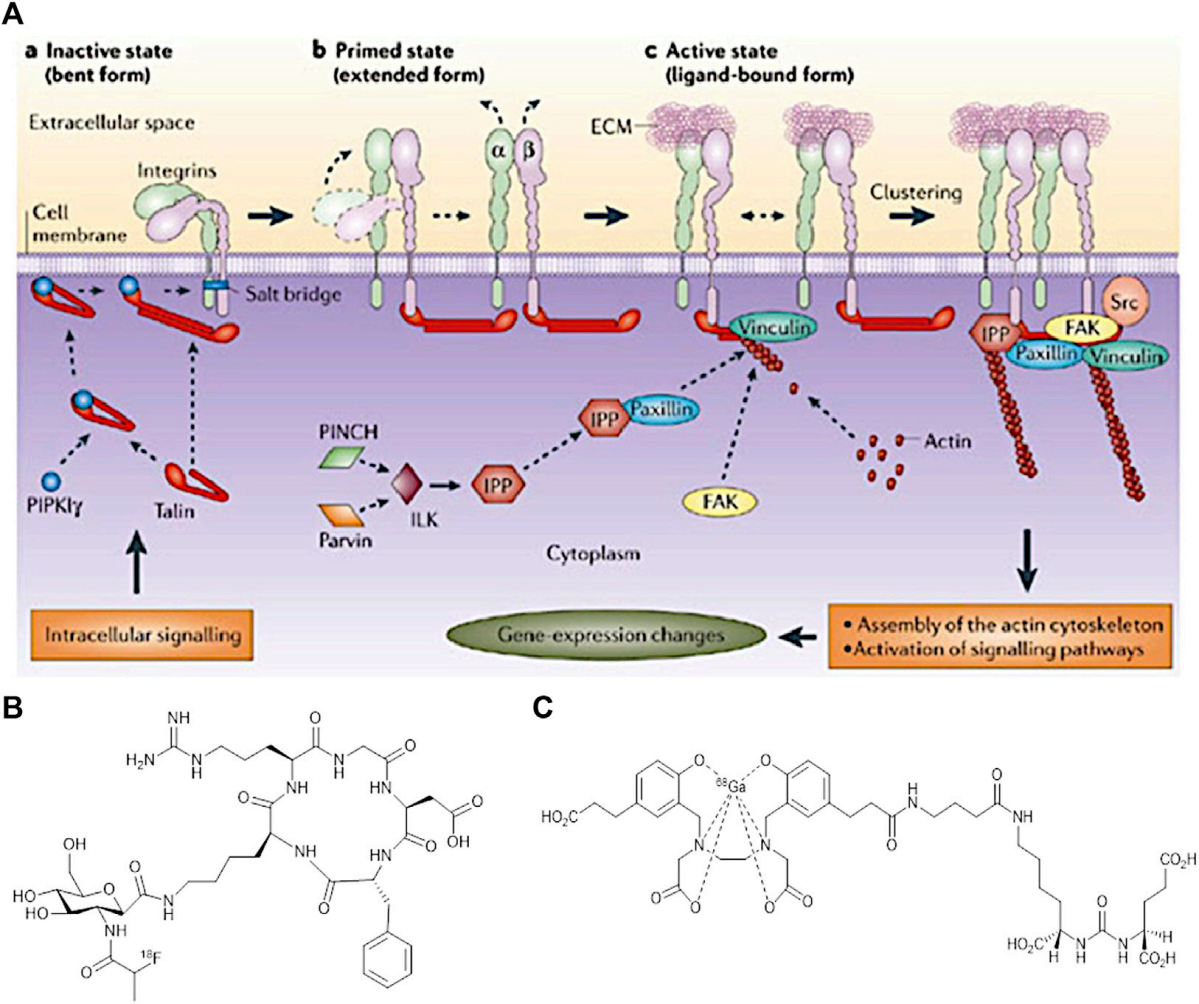
**(A)** Interaction of integrins with extracellular and intracellular molecules (Backer and Backer, 2012) **(B)** Structure of [^18^F]Fgalacto-RGD PET Radiotracer, and **(C)** The structure of [^68^Ga]GaPSMA-11. (Figure adapted from reference (Rudin, 2005)).

In association with the main aim of theragnostics, a specific and personalised treatment for a patient, research development has led towards the use of biomarkers. They can provide an early diagnosis that will enhance imaging techniques and cancer therapy to a large extend. According to literature, a biomarker can be a cell, a protein, an enzyme and even a cell membrane receptor. The activity of a biomarker could be recorded using imaging approaches to monitor a variety of biological processes occurring in the human body (Smith and Smith, 2012; Bernsen et al., 2015; Shi et al., 2016). The rapid and uncontrolled growth of tumour cells is known as an angiogenesis process, in which a specific type of blood vessels growth to provide the required nutrients and oxygen for further cancer cell development. In this sense, research studies have been performed for specific imaging of angiogenesis, mainly in preclinical studies, as the main focus is on diagnosing cancer, as it plays a crucial role in deciding upon the type of therapy. An example of an angiogenic biomarker is integrin α_v_β_3_, which is a transmembrane receptor involved in the Extracellular Cell Membrane interactions (ECM) (Wang et al., 2013). This integrin is overexpressed in tumour sites and therefore attracted immediate attention by clinicians. Cell adhesion and migration along the bloodstream is regulated by this type of integrin (Shi et al., 2016). Since, integrins have the ability to recognise a variety of specific sequence of peptides, they can be used for imaging the aggressiveness of cancer. Figure 7 shows the interaction of the integrin with extracellular molecules (Backer and Backer, 2012).

To illustrate the utility of α_v_β_3_, an Arginine-Glycine-Aspartic acid (RGD) is used, a peptide chain that is found to be present in many cancer sites. Its role varies from cell adhesion to proliferation. RGD binds specifically to α_v_β_3_ integrin and this specific binding can induce several changes in the tumour cell behavior and for that reason RGD could be conjugated with a drug or carrier molecule to be used as an imaging agent (Bernsen et al., 2015). The cyclic form of RGD (cRGD) resulted to a higher activity than its linear form. This could be expected since the cRGD is more rigid and so favour a stronger binding to the receptor site.

Imaging resolution and sensitivity is improved further by conjugation of RGD with radioisotopes. According to cell engineering these could be better potential anti-cancer agents (Backer and Backer, 2012). For instance, the discovery of the first [cRGD] PET radiotracer [^18^F]Fgalacto-RGD consisted of a sugar amino acid sequence conjugated with cRGD. Figure 6B shows this PET radiotracer which was very promising to show a faster detection of cancer in the previous years. However, it was proved that it was not that efficient in specific organs like liver, spleen and kidneys. Cell receptors in tissues of these organs show to have a very low RGD uptake and due to a high background to noise ratio, imaging could not differentiate between a tumour and a healthy cell. Future repetitions using multimeric cRGD peptides with radioisotopes could enhance the binding affinity in these human body areas (Chen H. et al., 2016).

Prostate Specific Antigen (PSA) is one of the first biomarkers used for PCa progression imaging. PSA is an active serine protein enzyme that is found in the endothelial cells of the prostate gland. It can exist either in its free form (_f_PSA) or as a complex. Monoclonal antibodies can be functionalised on the surface of a tumour tissue and recognise this motif; for example, the monoclonal antibody denoted 5A10 specifically binds to the surface of PSA. A variety of limitations in detecting and imaging PSA directly have been shown to lead to over-diagnosis (Ulmert et al., 2012). Due to the instability of PSA as a result of an equilibrium between its free and complexed form *in vivo*, its usage as a biomarker is now being limited for PCa diagnosis, despite being one of the first US Food and Drug approve (FDA) biomarker. A conjugate mAb and radioisotope PSA agent like ^89^Zr-5A10 however showed a higher contrast imaging of the tumour cells and a very low uptake by healthy tissues. Further analysis is required to understand the activity of this biomarker, but very promising results are expected as it can offer precise staging of cancer in a pre-clinical phase (Ulmert et al., 2012; Saini, 2016).

Another example of relevance to PCa detection is concerned with Prostate Specific Membrane Antigen (PSMA) targetting which is a transmembrane glycoprotein believed to be linearly correlated with PCa stages. The structure of PSMA (Figure 6C) consists of three different fragments each with differing number of amino acids in its composition. Earlier research suggested that PSMA is a powerful target for diagnostics of PCa (Chang, 2004; Nguyen et al., 2019) and as such this biomarker has emerged as an attractive imaging target due to high over expression at all stages of cancer. Challenges in meeting its therapeutic prospectives are yet to be overcome to explore its potential to delay further cancer development and metastasis (von Eyben et al., 2018). A characteristic imaging can result by the specific internalization of PSMA as an antibody attaches to it (Bouchelouche et al., 2010a).

Potential molecules that target PSMA can also be radiolabeled for a theranostic purpose. Additionally, significant PSMA-targeting work using ^64^Cu labelling of chelators has been reported and it reached preclinical investigations stages (Cui, et al., 2017). Detailed structure-function relationships were derived for the radiolabelling of a wide range of chelator-bombesin conjugates with ^64^Cu. ^64^Cu-labeled inhibitors of PSMA, which are based on the lysine−glutamate urea scaffold and supported on the macrocyclic chelators: NOTA, PCTA, Oxo-DO3A, CB-TE2A and DOTA. A range of bombesin conjugates in this series have been evaluated from the perspective of their pharmacokinetics as a measure of their relative suitability for or *in vivo* PET imaging with ^64^Cu (Banerjee, et al., 2014). In all cases, the radiochemical incorporation exceeded 60% and the purity was above 95%. Positron emission tomography (PET) imaging studies confirmed the accumulation in PSMA-expressing xenografts (PSMA + PC3 PIP) relative to isogenic control tumor (PSMA− PC3 flu) and background tissue. These extensive preclinical investigations highlighted the favorable kinetics and high image contrast provided by the bombesin conjugate of [^64^Cu]-CB-TE2A chelator, which was assigned to the higher stability of the [^64^Cu]CB-TE2A scaffold with respect to loss of free _64_Cu *in vivo* when compared with the other complexes from the series investigated (Banerjee, et al., 2014).

However, the challenge to this targeting approach is that PSMA can also be expressed in other types of cancer tissue like, colon and thyroid cancers (Chang, 2004). A variety of anti-PSMA mAb attempt to identify PSMA for binding and recognition of cancerous tissues with the first ever mAb denoted 7E11- ProstaScint. This attempt was not that successful as PSMA internalized before the mAb reaches the binding site (Chang, 2004; Bouchelouche et al., 2010a). Newly developed imaging advances have suggested that radiopharmaceutical agents with a lower molecular weight like [^68^Ga]GaPSMA-11, will be more dominant than radiolabeled mAbs (Bouchelouche et al., 2010a; Li et al., 2012; von Eyben et al., 2018).

A significant amount of PET imaging for prostate cancer diagnosis is currently being carried out using gallium-68 labelled small peptide molecules based on ureas that target the prostate-specific membrane antigen. The use of small urea peptidomimetics allows for a facile synthesis and rapid radiolabelling when conjugated to a gallium chelator such as DOTA or HBED-CC. The latter, denoted ^68^Ga-PSMA (Figure 6C), has had a big impact in clinical trials showing high specificity, usefulness in recurrent cases and successful localisation of metastases and is en route of becoming a standard in prostate cancer management in the clinic (Cortezon-Tamarit et al., 2019). ^177^Lu-PSMA is the theranostic pair for prostate cancer treatment. ^177^Lu-PSMA therapy is used for prostate cancer that has spread throughout the body and has become resistant to other treatments. Although these hard-to-treat cancers can’t be completely eradicated, ^177^Lu-PSMA therapy aims to reduce the size and progress of the cancer, ease symptoms and, in doing so, maintain or improve quality of life (Weineisen et al., 2015; Plichta et al., 2021).

### 3.4 Thiosemicarbazone-Based Metal Complexes as *Cancer* Theranostics

**FIGURE 8:**
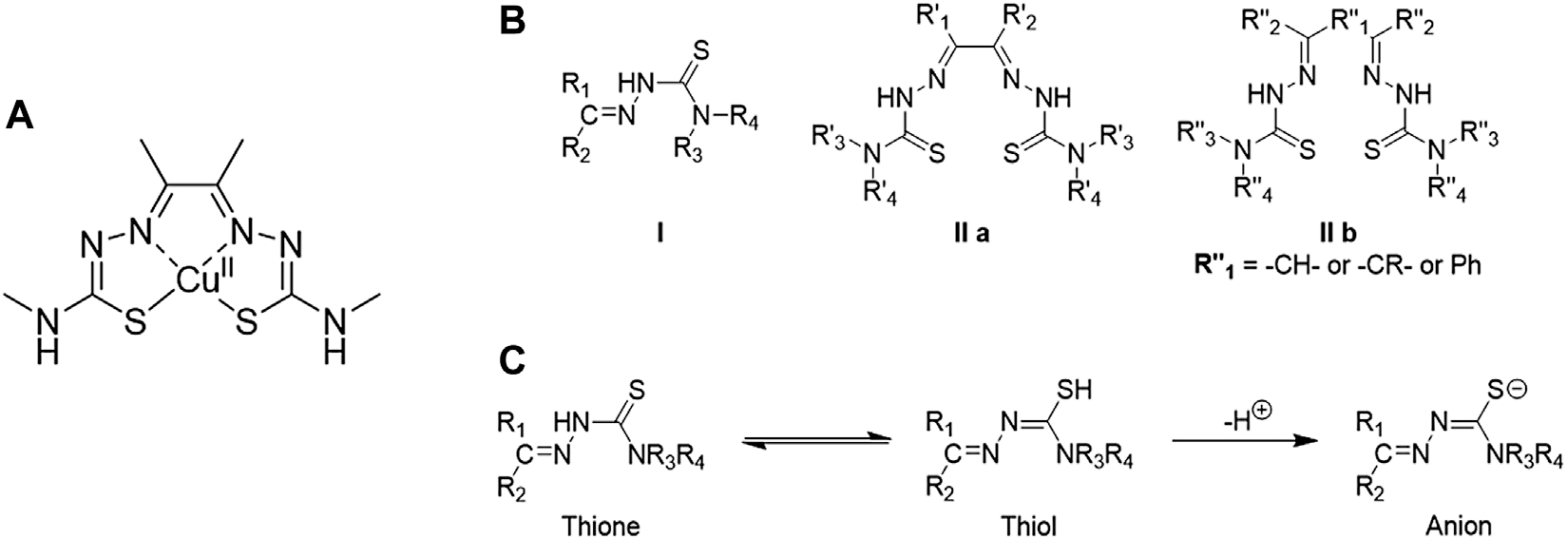
**(A)** Structure of diacetyl bis(N4-methylthiosemicarbazonato) copper (II), well known as [Cu(ATSM)], **(B)** Structural representation of common TSC backbones: mono-TSC (I), non-bridged *bis*-TSC (IIa) and bridged TSC (IIb), **(C)** Thione-thiol equilibrium of TSC which can lead to the anionic form.

Thiosemicarbazones (TSCs) have become important molecular building blocks for the development of novel biosensors (Seven et al., 2013; Yildirim et al., 2014), imaging probes and therapeutic agents. One of their main characteristics is the ease of synthesis and functionalisation which have been interested a lot of researchers worldwide to find biological applications. These ligands have also been explored for their ability to complex different metal cations which can improve other characteristics such as cytotoxicity, fluorescence emission or biocompatibility. One of the most representative examples of TSC is diacetyl bis(N4-methylthiosemicarbazonato) copper (II) ([Cu(ATSM)]) (Figure 8A). Such TSC metal complex has been employed as therapeutic agents to recover the copper imbalance caused by neurodegenerative diseases such as Alzheimer’s (Paterson and Donnelly, 2011) and represents the first case of thiosemicarbazone complex in clinical trials. This complex has become the starting point for researchers worldwide to synthesise and study either different ligands or metal cations for therapeutic and imaging applications.

**FIGURE 9:**
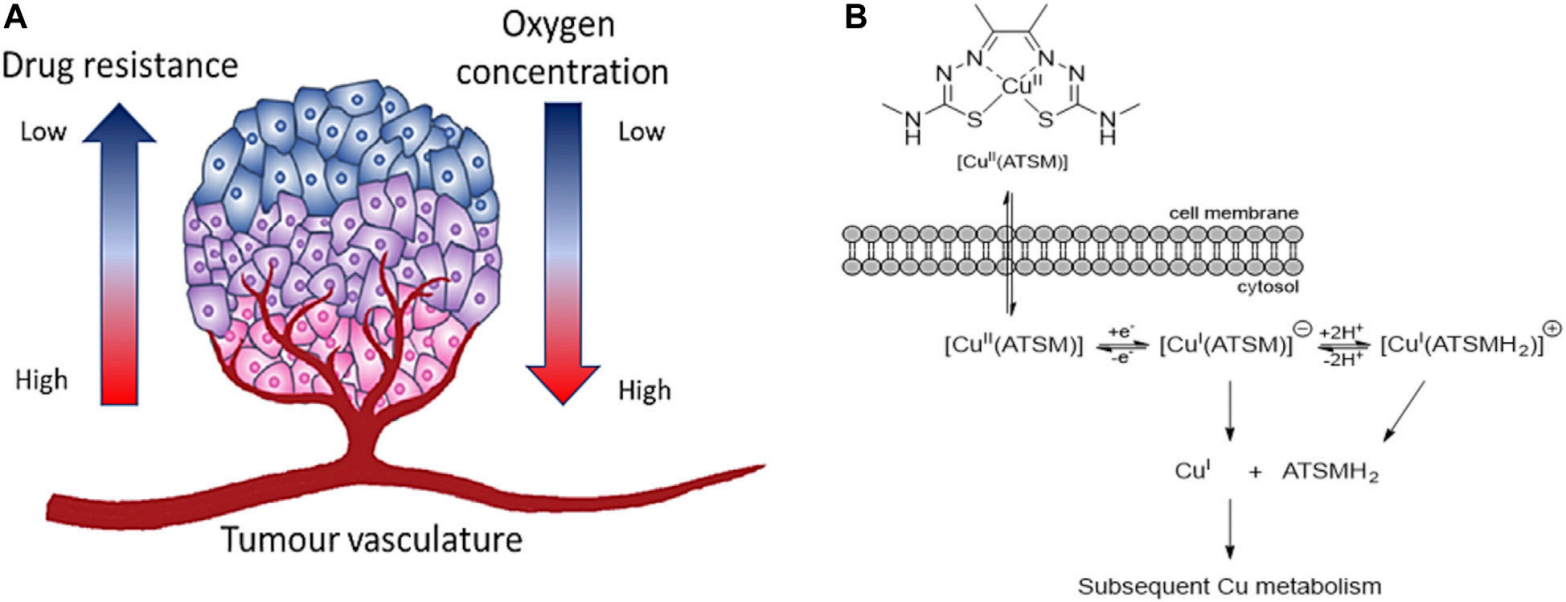
**(A)** Microenvironment of hypoxic tumour. The oxygen supply becomes restricted in cells once the rate of growth of tumour cells is progressing. Cancerous cells in hypoxic conditions can develop aggressiveness, metastasis, and resistance to therapeutic agents (figure adapted from Sharma et al. (2019)), **(B)** Schematic representation of the uptake of [Cu(ATSM)] and release of Cu(I) ions in hypoxic conditions (figure adapted from Challapalli et al. (2017)).

TSC ligands were discovered in the 1950s (Lobana et al., 2009). From the 1970s, their interest in synthesis and functionalisation started to increase, leading to their metal-TSCs complexes chemistry in the late 1980s.(Lobana et al., 2009). From the 1990s, the studies of TSCs as a free ligand or in metal complexes considerably raised and a lot of characteristics have been highlighted from their crystal structures, biological applications and optical properties. Two main class of TSC ligands can be distinguished. Mono-TSCs present different substituents on their backbone (R_1_, R_2_, R_3_ and R_4_ indicated in structure **I** Figure 8B) which can be alkyl, aryl or heterocyclic groups. On the other hand, bis-TSCs present two symmetric, or dissymmetric, arms which can be also bridged (R^″^_1_ in structure **IIb** Figure 9) by a C-C bond or an aromatic ring. Both *mono*- and *bis*-TSCs ligands can generate a thione-thiol equilibrium which can bind a metal cation in neutral form. In addition, after the loss of a proton, either in the amino or thiol groups, TSCs can complex metal centres in anionic form (Figure 9) (Lobana et al., 2009) The presence of donor atoms, such as nitrogen and sulphur, in the TSCs backbone, confers a wide range of binding modes of metal cations (Lobana et al., 2009). This characteristic has been explored with different transition metals such as copper (West et al., 1993), zinc (Antholine et al., 1977), cobalt (West et al., 1999), iron, (Walcourt et al., 2004), manganese (Othman et al., 1996), gold, (Abram et al., 2000), gallium (Kowol et al., 2007), ruthenium (Basuli et al., 1997) and many others.

In particular, TSCs metal complexes have been widely studied as therapeutic agents because of their cytotoxicity and biocompatibility (Dilworth and Hueting, 2012). In recent years, TSC derivatives with Zn(II) have been proposed as cytotoxic agents able to localise in the lysosomes and trans-metallate with Cu (Stacy et al., 2016). In addition, nickel (II) complexes of TSCs have found different applications such as antiretroviral agents for HIV (Stacy et al., 2016), antimicrobial drugs (Pelosi et al., 2010) and can induce apoptosis in different cell lines (Akbari et al., 2019).

Another characteristic which becomes important for TSCs metal complexes is the ability to be selective for “hypoxia”. Hypoxia is a condition which indicates the lack of oxygen in a region of the body or the entire body which was discovered by Thomlinson and Gray in the 1950s (Balachandran et al., 2018). The concentration of oxygen in normal tissues is higher closest to a blood vessel which gradually declines with the distance from the vessel (Figure 9A). In cancerous tissues, this concentration drops drastically near zero (Semenza, 2010) and cells can adapt to this condition because of hypoxia-inducible factor-1 (HIF-1) (Lu and Kang, 2010). HIF-1 develops responses that make cells to survive under hypoxia. These processes can promote the invasion, growth and metastasis of cancer cells in tissues and organs (Semenza, 2007).

In the past, different therapeutic agents have been revealed ineffective as the resistance and aggression of cancer grow under hypoxia conditions (Sharma et al., 2019). In this regard, the main target of cancer research in the last decades is to find compounds which are able to target HIF-1 (Xia et al., 2012) and being activated by hypoxia condition (Wilson and Hay, 2011). [Cu(ATSM)] is one example of *bis*-TSCs which has shown selectivity for cells under hypoxia compared to normal cells (Palanimuthu et al., 2013). Such hypoxia selectivity is due to its irreversible reduction of Cu(II) in Cu(I) in lack of oxygen which traps the monovalent ions inside the cells (Palanimuthu et al., 2013). At high concentration of oxygen, Cu(I) can be reoxidised in Cu(II) and be expelled from the cells (Figure 9B). Similarly, the couple cobalt (III)/cobalt (II) has been investigated with bis-thiosemicarbazone ligands as hypoxia-targeting complexes (King et al., 2017).

In recent years, radiopharmaceuticals have been playing an important role in cancer diagnosis and therapy (Dearling and Blower, 1998; Volkert and Hoffman, 1999). These agents are designed molecules which incorporate radioisotopes of specific atoms able to emit doses of ionising radiation to cancerous cells, able to kill them. Considering the radiation emission of radioisotopes, such radiopharmaceutical can be also used for imaging for example for PET. Among radiolabelled compounds, copper (II) *bis*-thiosemicarbazones, labelled with ^60^Cu, ^62^Cu and ^64^Cu, have increasingly considered valid agents for *in vivo* imaging in hypoxic tissues (Dearling et al., 2002).

This interest has raised thanks to the simple coordination and redox chemistry of copper to bisthiosemicarbazones, its biochemistry and metabolism *in vivo*, (Vāvere and Lewis, 2007). In addition, the isotopes of copper, such as ^60^Cu, ^62^Cu and ^64^Cu, present versatile positron decay schemes (^60^Cu: half-life (t_1/2_) = 0.40 h, positron emission (β^+^) = 93%, electron capture (EC) = 7%); ^62^Cu: t_1/2_ = 0.16 h, β^+^ = 98%, EC = 2%; ^64^Cu: t_1/2_ = 12.7 h, β^+^ = 17.4%, EC = 43%) (Vāvere and Lewis, 2007) which have been revealing to be important for PET imaging.

As stated above, copper-64 has been extensively used in PET imaging due to its long half-life, long enough to allow the distribution of this nuclide from the production centre to the imaging centre without a cyclotron (Alam et al., 2016). These isotopes can be produced using reliable, reproducible and commercial production systems, however access to radio-copper diagnostic imaging procedure or therapies are available only in a relatively small number of medical centres (MRC, 2017).

In the last decades, gallium-68 has become a valid alternative to copper-64 thanks to its shorter half-life (t_1/2_ = 68 min) (Alam et al., 2016) and its potential use as a therapeutic and imaging agent under hypoxic condition (Alam et al., 2016). Despite its different coordination chemistry, ^68^Ga has been incorporated to thiosemicarbazone derivatives (Chan et al., 2010; Al-Hokbany et al., 2014; Alam et al., 2016) which has shown an increase of activity of TSCs ligands (Chan et al., 2010). In addition, gallium-68 TSC complexes can be used in fluorescence *in vivo* imaging because of the radioisotope of Ga is diamagnetic and do not interfere with fluorescence emission, which copper-64 complexes suffers.(Chan et al., 2010). This dual-modality of fluorescence and positron emissions of ^68^Ga has brought researchers to increase the range of metal radioisotopes useful in fluorescence and PET imaging. Indium-111, zirconium-89 and technetium-99 m are other examples of radioisotopes have been using in both fluorescence and PET imaging (Thorp-Greenwood and Coogan, 2011). However, these radioisotopes require specific generators and sometimes the production centre can be far from the imaging centre. Considering such problems, different fluorescent TSC metal complexes have been introduced as an alternative to radiolabelled compounds. In recent years, the versatility and ease of the synthesis of these TSC metal complexes have been widely explored for their optical properties, cytotoxicity and biocompatibility (Cortezon-Tamarit et al., 2016). Most of these metal complexes present fluorescence emission in different ranges of wavelengths which can be modulated by introducing fluorophores linked to the backbone (Cowley et al., 2005; Li et al., 2010; Kate et al., 2014) or being part of the backbone (Aina et al., 2007; Arrowsmith et al., 2011).

Considering the potential use of thiosemicarbazones as therapeutic and imaging agents, in the last decade, different research groups have explored the combination of TSC metal complexes and the fragments of bombesin for targeting specifically GRPR and able to exploit either therapy or imaging (Hueting et al., 2010; Paterson et al., 2010; Paterson and Donnelly, 2011). Novel fluorescent imaging probes have been reported in the last 10 years which present the targeting unit of bombesin peptide to detect the GRPRs on the prostate cancer cells and image them in different fluorescent techniques (Lee C.-M. et al., 2010; Shrivastava et al., 2014; Sturzu et al., 2014; Tseng et al., 2017). These fragments of the bombesin peptide need to be anchored to a fluorescent scaffold to be detected by fluorescence methodologies because of such peptide are weakly fluorescent to be imaged accurately.

As previously mentioned, radioactive compounds can be still considered a risk for the patients’ health and moreover, imaging methods such as PET and MRI can results expensive to patients, require sophisticated instruments and specialised medical personnel. Early-stage detection of the occurrence of cancer *via* fluorescence imaging has become a fast, sensitive and low-cost alternative to PET imaging. In the last decades, fluorescence methodologies have become important tools to investigate biological processes and detect cancerous cells thanks to their sensitivity, low-cost and ready-to-use equipment. The use of fluorescent methodologies has increased the design and synthesis of fluorescent molecules which can also target hypoxic tissues or specific biomarker in cancerous cells, of relevance to PCa targeting.

## 4 Applications of Nanoparticles in the Molecular Imaging of Prostate *Cancer*

*Cancer* nanotechnology approaches promise to open up the possibility of more personalised and specific cancer targeting for diagnosis as well as therapy. Upon its introduction in biomedicine nanotechnology has converted an anatomical structure imaging into a molecular imaging of a tumour tissue (Cai et al., 2008). Combining the synthetic advantages of NPs with the available imaging techniques could be very successful towards achieving high selectivity and a targeted imaging strategy. For example, gold NPs (AuPs) are widely used, not only because they are non-toxic but also due to their specific physical and optical characteristics. Their applications vary from radiotracers in PET/SPECT to contrast agents in CT. A key point that differentiates AuPs from other NPs is their multi-functionality, including the ability to incorporate other metal tags or be conjugated with small molecular units (Bulte and Modo, 2016). Specifically, AuPs are used to reduce the limitations of the CT method by differentiating soft tissues with similar structures. The high atomic number of gold rendered it an efficient contrast agent that provides both higher contrast images and less radiation exposure to patients.

An additional feature of AuPs is their ability to attach to the transendothelial pores of a cancerous tissue for specific and targeted imaging. Their small size enables them to accumulate on tumour sites more than in healthy tissues because of the EPR effect discussed previously. Conjugation of AuPs with molecular ligands such as an antibody, a peptide sequence and even DNA permit a well monitored targeted imaging (Cai et al., 2008).

The specific interactions of a peptide chain with a cell membrane receptor can increase the binding time of such contrast agents and therefore offer cancer diagnosis based on biomarkers. Research suggests that the use of gadolinium loaded dendrimer entrapped gold NPs(GdeAu DENPs) was found to be very supportive in imaging heart tumour tissue when using a hybrid CT/MRI (Bulte and Modo, 2016).

The terminology nanodiagnostics generally refers to the use of NPs for clinical diagnosis of a disease. This field is very capable especially for the early detection and diagnosis of PCa, an adenocarcinoma of the prostate in male population. QDs have the prospective to develop further already existing imaging and detection approaches (Peng and Li, 2010). Quantum dots (QDs) are engineered fluorescent nanoparticles with unique optical and chemical properties, which have shown a great potential as promising platforms for biomedical applications (Mirzaei et al., 2017). These semi-conductor nanocrystals with a several nanometres diameter have specific physical and chemical properties. They attracted enormous attention since they can emit light of a single wavelength ranging from ultra violet (UV) to near infra-red (IR) spectrum. As a result, this feature can be used to categorize different types of QDs based on their size and shape. Distinct features of QDs enable them to provide high selectivity and sensitivity when a NP based agent targets a tumour site. These features comprise of a single wavelength excitation, strong light absorbance and lastly their easy modification to be biocompatible in human body tissues (Azzazy et al., 2006). For instance, CdSe/ZnS QD is frequently used and tested in many clinical diagnostic applications of PCa. This QD is solubilised by functionalisation of its surface by water soluble ligands to allow *in-vivo* imaging. Additionally, bioconjugation of CdSe/ZnS QD with specific biomarkers of PCa including PSA and PSMA, enables a huge increase in sensitivity of a targeted imaging. Multi-detection can be reached due to a variety of colours of QDs being excited by a single wavelength, resulting to an earlier diagnosis (Peng and Li, 2010).

It has been demonstrated that QDs-based agents for cancer imaging could be very effective in laboratory settings and pre-clinical applications. However, argument has arisen about the safety issues involving their use in patients, which hampered their progress to clinical trials. One of the main concerns is their composition, as most QDs consist of heavy and toxic metals like Cd^+2^. Other studies suggest that there is no correlation of the amount of a heavy metal with the resulting toxicity and that other factors such as size of NP, composition and concentration of its coating should be considered (Azzazy et al., 2006; Peng and Li, 2010).

Overall, the applications of NPs in biomedicine are in infancy but these have been highlighted as potentially useful agents for the molecular imaging of tissues and treatment of non-communicable diseases such as cancer. The plethora of endless shapes and types of NPs with different physical and electronic properties could make them very helpful diagnostic agents (Azzazy et al., 2006). New technology may be able to overcome the current challenges that QDs possess with respect to higher sensitivity for diagnosis. Additional clinical trials are also required to understand better the health and safety facts of QDs and aim towards diminishing them.

### 4.1 Targeting Prostate *Cancer* Using Nanotechnology Tools

Nanotechnology is an emerging platform in the biomedical field that allows the interaction of nanomaterials with biomolecules at a molecular level (Saini et al., 2010). Nanotechnology studies the methods aiming at controlling matter on the atomic and molecular level, and involving particles with diameters within 1–100 nm in at least one dimension. Drug delivery systems, tissue engineering and molecular imaging are some of the areas where nanotechnology made a huge impact e.g. with the development of theranostic agents for cancer (Satalkar et al., 2016). In this sense, the chemical modification and encapsulation of NPs permitted their selective penetration into tumour tissues and allow them to target specifically abnormal sites for both an early detection and halt their uncontrolled growth (Sandhiya et al., 2009).

Recent improvements in cancer nanotechnology established better imaging methods for specific detection and targeted therapy of Prostate *Cancer* (PCa). Imaging cancer using targeting biomolecules is an operative plan to detect specific tumour sites and provide a better patient management for deciding the appropriate therapy. Theragnostic agents are designed in way that host both diagnostic and treatment abilities that can reduce the mortality rate of PCa, what is known as nanotheragnosis.

**FIGURE 10:**
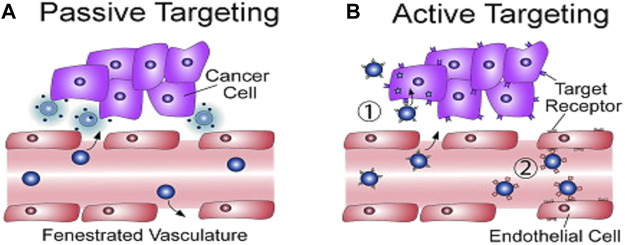
Schematic comparison between **(A)** Passive and **(B)** Active Targeting (figure adapted from reference (Wicki et al., 2015)).

Overall, there are two ways to target a tumour cell, either by passive targeting or active targeting. Figure 10 shows the two different targeting pathways (Wicki et al., 2015). Vascular Endothelial Growth Factor (VEGF) shows to be essential for the progression of the disease as it regulates the formation of blood vessels to provide tumour sites with nutrients for further growth. The accumulation of these agents on surface of permeable abnormal blood vessels of tumour tissues *via* the Enhanced Permeability and Retention Effect (EPR) is known as the passive targeting. However, there are many limitations to be overcome during such a targeting method, such as the heterogeneous surface of cells preventing the cellular uptake of the nanocarriers and respectively their binding affinity. To avoid such restrictions scientists have introduce active targeting.

This approach involved the use of biomolecules, like proteins or antibodies, to modify the surface of the nanocarrier agent for a specific binding of these molecules on receptors found on the cell membrane of tumour cells. Active rather than passive targeting is considered more advantageous as the controlled pharmacokinetics and biodistribution of a NP facilitates the higher cellular binding and reduced uptake by healthy cells. This way, retention time of a nanocarrier for imaging and diagnostic purposes it is expected to be enriched (Williams et al., 2013).

Recently, nanoparticles have become widely explored for their nanomedicine applications and a significant research effort involved the design of targeted NPs for drug delivery systems. This has promoted the emergence of a new field known as nanomedicine. Nanoparticles can be classified in a wide range of categories based mostly on their size, shape, physical and chemical properties. These factors play a major role on their applications as synthetic platforms for medical and bioimaging for disease diagnostics and therapeutics (Khan et al., 2019). Studies have shown that the size, shape, assembly and surface functionalisation of a NP influence the specificity of its role. What attracted enormous attention is the high surface area to volume ratio that NPs have, allowing their utility in many fields, including physics, engineering and electronics (Nune et al., 2009). In general, the structure of these nanoparticles as drug delivery systems is composed of three layers:

1) The core, which is the central part of the nanoparticle.
2) The shell layer, mainly a chemical coat different form the core.
3) The surface layer, can be readily functionalized with a variety of small molecules like polymers, metals or even peptides (McKittrick and Shea-Rohwer, 2014).

### 4.2 Sensing and Detection Platforms

Whilst some of the current PCa diagnositic methods were found to have lower false-positive rates than PSA screening, they are also significantly more time-consuming with low success rate in their ability to detect small metastases or small primary tumors. Therefore, the development of new, improved screening methods is an ongoing endeavor and various new approaches have been developed aiming to stratify the patient population in terms of surveillance, indolent vs aggressive PCa and to monitor treatment and progression of the disease. The success of this process can only be ensured through the accurate measurement, followed by monitoring of host biomarkers. Biosensing approaches could provide response data which will inform prognosis, thus allowing the stratification of patients. This can also provide a prognosis of the treatment response data which will allow an enhanced confidence in the patients’ selection to treatment in earlier phases of the disease, and an earlier diagnosis.

Regarding current synthetic approaches of nanoparticle biosensors designed specifically for PCa, several materials have been proposed, quantum dots, gold nanoparticles and graphene oxide being some of the most widespread in terms of clinical trials and applications. Some examples have been chosen from literature, each using different techniques and materials and are described below.

#### 4.2.1 Quantum Dots as Biosensing Tools

Quantum Dots have unique optical properties that allow them to deliver drugs to targeted cancer cells very selectively and efficiently. Their fluorescent nature means that they can be easily monitored throughout the body. They can be used for the monitoring of proteins, DNA, anions, small molecules and metal ions. The main challenge concerning their development and use is the design of sensors that have a high selectivity, sensitivity and stability (Malam et al., 2009).

**FIGURE 11:**
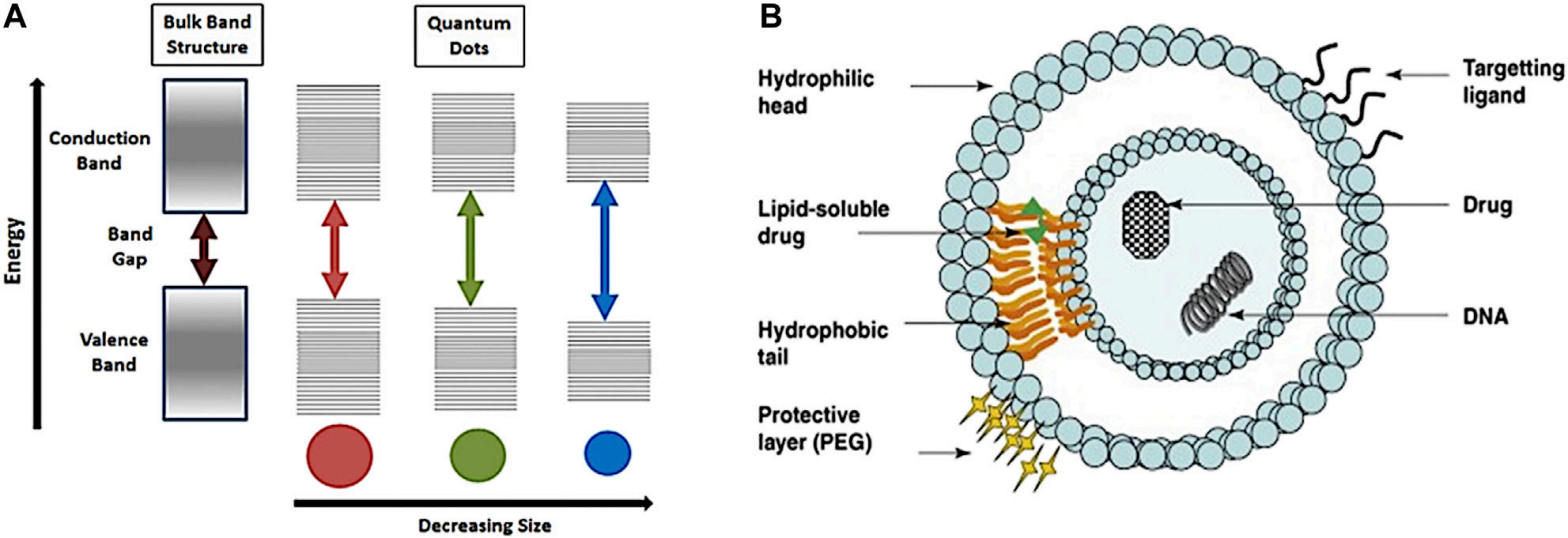
**(A)** Electronic band gap of QDs with varying particle size. (Figure adapted from reference (McKittrick and Shea-Rohwer, 2014)), **(B)** Protected liposome structure by PEG polyether. (Figure adapted from reference (Malam et al., 2009)).

Their semiconducting nature permit electrons to get excited from the valence band to the conduction band upon absorption of light. Fluorescence can occur when the excited state electron returns to its ground state, emitting a photon with a particular wavelength. Smaller QDs have a larger band gap as shown in the electronic structures in Figure 11A. Therefore, their photon emission can be monitored during their synthesis and adapted according to the desired use. The availability of a wide range of structures comprising of conjugated and functional QDs enables their application to be viable in bioimaging, solar cells and light emitting devices (McKittrick and Shea-Rohwer, 2014).

Apart from this, creating hydrophilic QDs allow a variety of clinical applications to be performed, one of which is tumour cell identification. Locating cancer using CdSSe/ZnS QDs which are designed using a CdSSe inner core encapsulated within a ZnS outer core, showed to be effective fluorophores with a long fluorescence lifetime. Hence, allowing them to target and locate individual cancer cells for faster diagnosis. QDs have created a huge impact in molecular imaging and further advances on the techniques to synthesise a more stable bio-conjugated QD with proteins, antibodies and even DNA attached will broaden their use in clinical applications (Probst et al., 2013).

The Zn^2+^ ion, being a component of chromatin, the material chromosomes are made of, plays a crucial role in DNA replication, transcription and repair (Puri et al., 2009). A healthy prostate cell contains a higher concentration of Zn^2+^ than a PCa cell. Therefore, Zn^2+^ chelation or Zn^2+^ administration is an important therapeutic approach for the regulation of PCa cells development and progression (Frangioni, 2008).

Quantum Dots involving cadmium are usually highly toxic with low sensitivity and stability (Scher et al., 2008). To overcome this problem, a carboxymethyl chitosan coating is added on the surface of CdTe QDs reducing their toxicity and enhancing their biocompatibility as well as endocytosis. It also provides for a strong binding surface for Zn^2+^ ions, which, as soon as they bind, activate the QDs’ photoluminescence effect.

The formation of CMC-CdTe QDs exploits the electrostatic interaction between amino groups in carboxymethyl chitosan polymer chains and the carboxyl groups of the QDs. The photoluminescence intensity of the CMC-CdTe QD probe is directly proportional with the concentration of Zn^2+^ ions when in the range of 5 × 10^−6^ M to 5 × 10^−3^ M.

Overall, this probe provides a good cell compatibility as well as adequate sensitivity and selectivity for intracellular Zn^2+^ sensing. It is really promising for the sensing, diagnosis and prognosis of PCa, as well as for assessing the effects of Zn^2+^ after therapy. Due to its positively charged surface, the probe is attracted and taken up by specific PCa cells (PC-3M), allowing for *in vitro* fluorescence imaging of the cancerous cells (Malam et al., 2009).

Biomarkers for PCa also include PSA (Prostate Specific Antigen) and ANXA3 (Annexin A3), the latter also being expressed in lung adenocarcinoma. PSA is not to be relied on for early detection of PCa since a high or low PSA value can be misleading. High PSA values are not common only in PCa but also in other prostate conditions. PCa patients can be found to have low PSA levels as well (Estelrich et al., 2015).

On the other hand, ANXA3 is a specific and non-invasive biomarker (Anderson and Welch, 1999; Bouchelouche et al., 2010b) that remains stable in urine samples for more than 48 h at room temperature. It is therefore more suitable for early diagnosis. CdS QDs coupled to Au nanoimmunosensors on a Quartz Crystal Microbalance (QCM) is an ultrasensitive and label-free way to detect ANXA3. It works over a large dynamic range with minimal interference from other proteins. This probe is assembled by functionalizing CdS QDs with–COOH functional groups and binding them to a cystamine self-assembled monolayer on the Au/QCM. Polyclonal anti-ANXA3 bonds covalently on the probe surface, which makes it easier for ANXA3 to be detected by the probe (Paterson et al., 2014).

Another example is CuInS_2_ QDs, which are water soluble, near Infra-Red emitting and highly efficient while also overcoming the toxicity and bio-compatibility problems that Cd-containing QDs cause under extreme conditions. These properties include: narrow, tune-able emission spectra, high quantum yields and simultaneous excitation of multiple fluorescence colours.

Anticancer drugs conjugate to CuInS_2_ QDs, which not only provide lower toxicity, but also higher *in vivo* drug retention times and improved solubility and targeting. Near-IR wavelength light has a strong tissue penetrating power, with low tissue scattering effects (Evans et al., 2018). These specific QDs also use aptamers to conjugate drugs and deliver them to the target cells. Aptamers are short, single-stranded oligonucleotide ligands, with high specificity and affiliation with a broad range of analytes, such as molecules, drugs, ions, proteins or cells (Berger, 2003). Quenching of the QDs’ fluorescence emission often signifies that the drug has successfully reached the target cells as described by X. Yan et al. in their work on near-IR fluorescent nanoprobes for enzyme-substrate system sensing (Jadvar, 2013). A probe for PCa cell detection can be formed by 3-aminobenzeneboronic acid (APBA) bonding covalently to the surface of the QDs. *In-vitro* imaging was used with PC-3M and it was observed that the probe could be found clearly in the medium (Evans et al., 2018).

Another QD biosensor can be developed based on the H_2_O_2_ emission from cancerous cells. H_2_O_2_ can be found in most cells in the human body and is involved in signal transduction processes. Low H_2_O_2_ concentrations in living cells are considered healthy, whereas high concentrations damage cells and can cause cell mutations. To achieve the necessary control over these signal transduction processes in which H_2_O_2_ is involved, its quantity must be determined: one of the best candidates for this are reduced Graphene Oxide QDs/ZnO hybrid nanofibers-based electrochemical biosensors (rGO QDs/ZnO hybrid NFs). Reduced GO QDs are well dispersed on the surface of ZnO NFs, giving a high electroactive surface area with high electrocatalytic activity toward H_2_O_2_ reduction. They also overcome the sensitivity, selectivity, stability and repeatability issues caused by nanomaterials using metals, metal compounds and carbon, measurements which were coming from Cd^2+^ or Ascorbic Acid stimulus, instead of from healthy or cancerous cells, thus making the results difficult to interpret. Use of the proposed rGO QDs/ZnO hybrid biosensors on PCa cells which are being treated with anticancer drugs demonstrated that the sensors can be applied successfully in drug screening (Imlimthan et al., 2018).

#### 4.2.2 Gold Nanoparticles for Biosensing Applications

Gold nanoparticles (AuNPs) have some unique properties that have made them attract a lot of attention over the last few years, most importantly their optical and self-assembly properties, as well as their biocompatibility, robust nature and large surface areas (Berger, 2003; Tegler et al., 2012). A few of the nanosensors described throughout this review use aptamers or antibodies. Even though they can be considered very similar at first, there is a simple way to tell them apart. Aptamers can be distinguished from antibodies by the fact that they can be easily produced and modified, and are sufficiently stable to be stored.

As mentioned formerly, high levels of PSA can be an indication of PCa, even though they can be misleading. The concentration of PSA in blood is usually very low, which means that a high-sensitivity method is required to detect it. One such method used recently is based on LSPR (Localised Surface Plasmon Resonance) (Kai, 2010). Antigens are used as receptors for PSA on the AuNP surface and as soon as this reaction takes place the refractive index of the medium changes, and can be detected using LSPR. A new method proposed in 2017 (Berger, 2003) uses the LSPR extinction spectrum of Au nano disk arrays, functionalized with DNA aptamers. As low a concentration of 2 nM of the aptamers is needed on the AuNP surface to achieve a linear relationship in the range 1.7 ngmL^−1^ to 20.4 ngmL^−1^. When a 20 nm thick Au nano disk array is used, LSPR is predominant with a molar extinction coefficient, ε, of 10^11^ Lmol^−1^cm^−1^. Changes over very short ranges on the sensor surface can be observed with this method, and the DNA aptamer functionalization increases the AuNP stability and improves its interaction with PSA. Finally, this method is label free, time efficient, simple and sensitive, with a high reliability (Berger, 2003).

ELISA (Enzyme-Linked Immunosorbent Assay) is a widely used method for detecting raised levels of prostate biomarkers, which could indicate cancer. However, detection through this method is only allowed when biomarker levels are too high, meaning the disease has progressed significantly. Keeping in mind that most types of cancer respond better to treatment the earlier they are diagnosed, a more sensitive method is needed for cancer sensing. One such method proposes a fluorescence-activated probe with a detection level two orders of magnitude lower than most conventional fluorescence probes.

**FIGURE 12:**
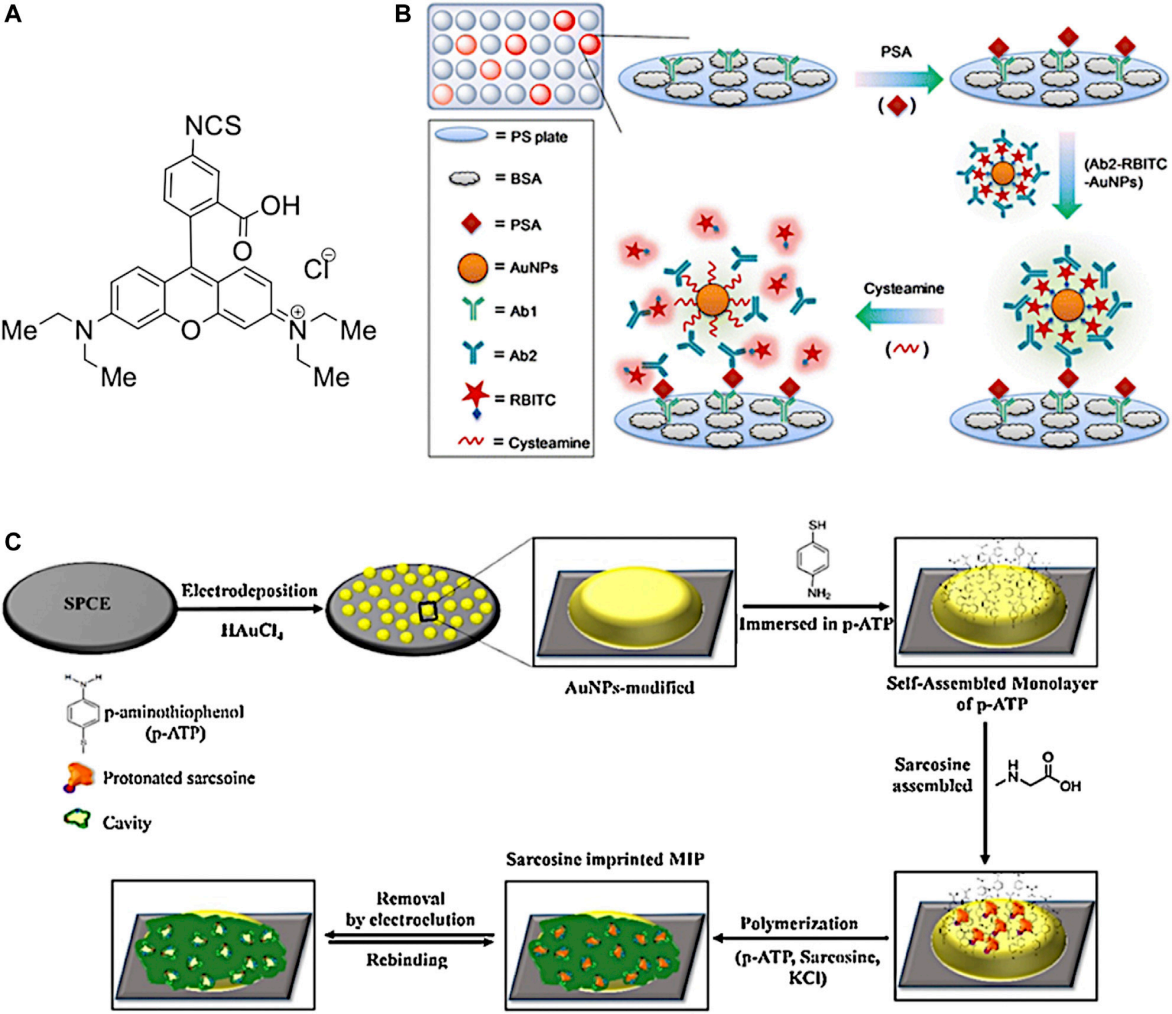
**(A)** Example of a fluorescent dye (denoted RBITC) used in the synthesis of the fluorescence-activated probe Ab2-RBITC-AuNP, **(B)** procedure as demonstrated by D. Liu et al. for the synthesis of the AuNP activated probe for early PSA detection (figure adapted from Ref (Liu et al., 2013)) **(C)** an overview of the procedure in described for the formation of MIP/AuNPs/SPCE (figure adapted from (Nguy et al., 2017)).

A normal fluorescence-activated probe consists of an acceptor (in this case AuNPs, which act as a quencher) and a donor (e.g. a fluorescent dye). The higher the loading efficiency of the dyes on the AuNPs surface, and the higher the quenching and unquenching abilities of the dye-AuNPs conjugates, the higher the probe sensitivity. In the example given, the dye used was RBITC (Rhodamine B Isothiocyanate Figure 12A), which not only is water-soluble and strongly fluorescent, but also effectively quenched by AuNPs. It is also very easily removed from the AuNPs surface by addition of thiol compounds in high concentrations. When the AuNPs are removed, the dye becomes unquenched.

The synthetic approach is as follows: RBITC is firstly conjugated on the surface of the AuNPs before it is covered with detection antibodies (Ab2) to form Ab2-RBITC-AuNPs. The two positively charged amine groups on RBITC form electrostatic interactions with the negatively charged Ab2. AuNPs are chosen as the donor due to their very high quenching efficiencies (up to 99.8%). Fluorescence is quenched via the NSET effect (Nanoparticle Surface Energy Transfer) and is recovered as soon as the dye is removed from the AuNPs surface. The intensity of the recovered fluorescence is proportional to the concentration of the biomarker in the patient serum sample used. A proposed biomarker that can be targeted is PSA, but this method can also be applicable to other PCa biomarkers; this method is low-cost and easily adaptable, something that can be of great value in settings where the infrastructure or medical expertise required for more advanced detection and treatment options are limited (Fass, 2008).

**FIGURE 13:**
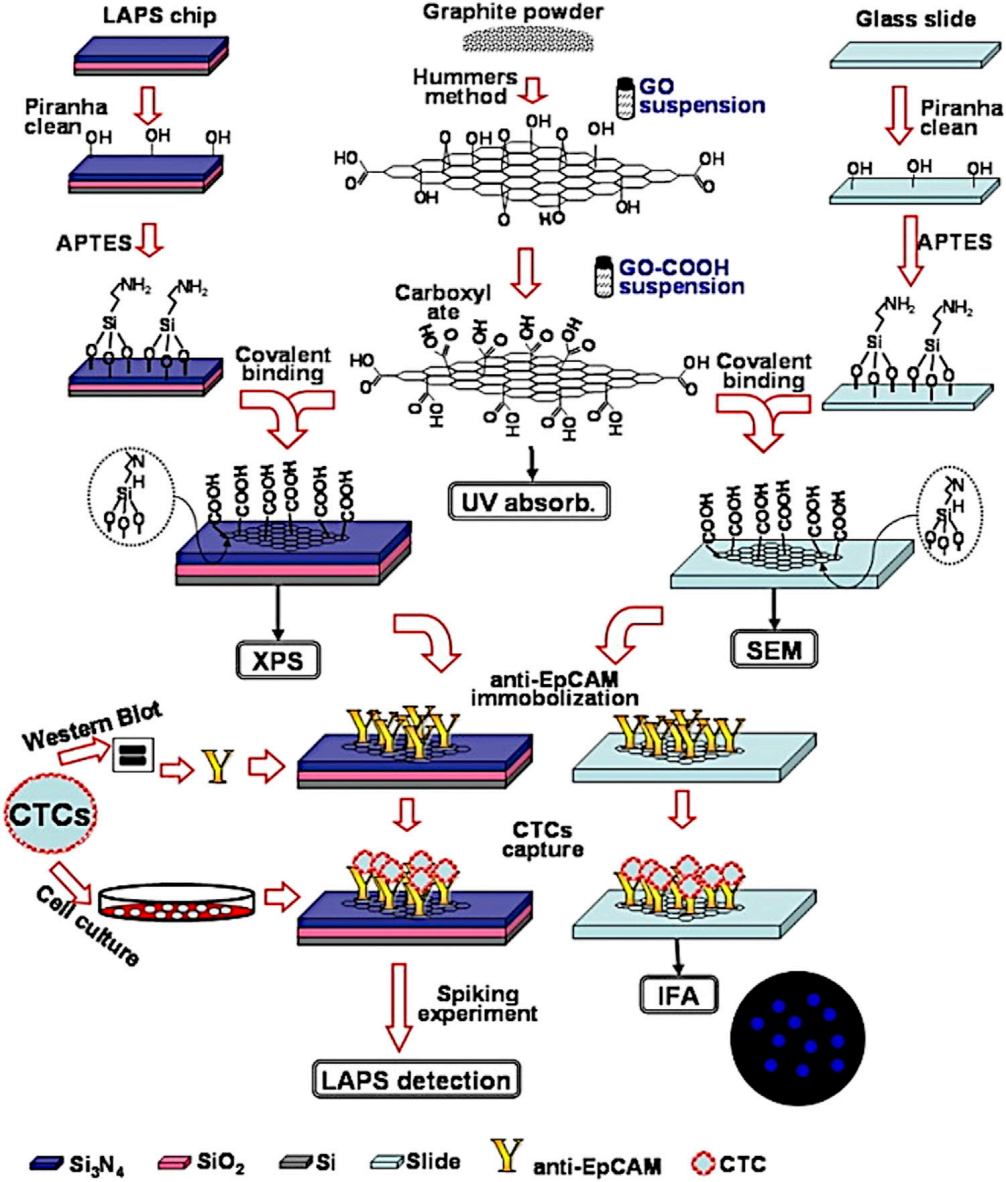
Overview of the synthetic procedure and analytical protocols for CTC-LAPS (Figure adapted from Ref (Gu et al., 2015)).

Sarcosine is a methylated derivative of the amino acid glycine and is considered as another PCa biomarker (Murphy, 2010). The potential it presents as a possible indication of PCa is large as it comes free of the disadvantages PSA presents (i.e., a raised PSA level may be misleading as it is not always an indication of prostate cancer). However, research argues that sarcosine is not a good PCa marker, but this may be due to its molecular mass being the same as alanine, resulting in MS not being able to tell them apart (Rudin, 2005). It can be found in blood, urine, as well as serum samples of patients suffering from metastatic prostate cancer and it can be measured using HPLC/MS or GC/MS. However, these methods are costly, as they require specialized equipment, whilst the resulting device prototypes are difficult to assemble and calibrate. Therefore, a new method for sarcosine detection and quantification would indeed be welcome in order to overcome the problems stated above. This method comes in the form of Screen-Printed Carbon Electrodes (SPCEs) modified with AuNPs *in situ* (Figure 13). The AuNPs are synthesised by electrodeposition on the SPCEs using Cyclic Voltammetry (CV). MIPs (Molecularly Imprinted Polymers) (Takayanagi et al., 2004) are used as synthetic sarcosine receptors on the AuNP/SPCE surface. More specifically, ultrathin 4-aminothiophenol layers where polymerized into MIP membranes with microscale morphology and high conductivity. The AuNPs cause the MIPs to grow homogeneously and increase their active surface. Overall, this sensor has a higher screening performance than previous methods used, such as SPAuE (Screen Printed Au-ink Electrodes) at all concentrations as well as more enhanced sensitivity and specificity comparable to natural receptor molecules (Yankeelov et al., 2016).

#### 4.2.3 Carbon-Based Nanomaterials and Nanoparticles for PCa Biosensors

Fullerenes, nanodiamonds, nanotubes and graphene are some of the carbon-based structures which have benefited of wide interest towards biosensing appications, as the initial assumption was that these particles can be used in humans with minimum risks (Cha et al., 2013). Graphene can be thought as the starting point on the formation of carbon nanoparticles (CNPs) since it consists of a thin planar sheet of sp^2^ bonded carbons. Carbon nanotubes (CNTs) based on graphene structure, can have a single or multi-walled cylindrical long carbon tubes, allowing them to have tunable physical features (Nune et al., 2009; Khan et al., 2019). Their high mechanical strength, electrical and thermal conductivity allows them to have a multi-functional nature. Additional advance on their mechanical strength can be enhanced by the rigidity and flexibility that their structure offers. There is significant research interst in the use of CNTs as drug delivers, cell labelling and imaging agents since their surface can easily be modified for specific targeted imaging or drug delivery. The hollow tubes of their structure acts as an active site for modification and to ensure a higher catalytic activity of a biomolecular process. A non-covalent modification though, may appear to be more effective as it can maintain the properties of the CNTs’ structure (Alim et al., 2018).

An example that applies to the sensing and detection of PCa, besides QDs and AuNPs is graphene oxide (GO), and attention will be given to it in this report, due to its unique properties and applications in the nanomedicine field. The planarity and large surface area of graphene allows for a wide variety of surface modifications. Graphene oxide (Bernsen et al., 2015) is only one example that is easy to produce and has some important applications in the sensing of PCa.

A possible application of GO is in the detection of CTCs (Circulating Tumour Cells) (Smith and Smith, 2012). These are cancer cells that are detached from the primary tumour and circulate the bloodstream. They can be detected in the early stages of cancer and could explain metastasis since a high concentration of CTCs is in strong correlation with an aggressive disease and increased chance of metastasis (Wang et al., 2013; Shi et al., 2016). However, these cells are very rare and due to this, they cannot be isolated easily. There are a few methods to detect CTCs (Connelly et al., 2016), but the only currently FDA (Food and Drug Administration) authorized platform for CTC monitoring is CellSearch, which uses complex machinery that is not only costly but also time-consuming and complicated to use (Connelly et al., 2016). This method uses anti-epithelial cell adhesion molecule (anti-EpCAM) magnetic beads for CTC capture, followed by staining to visualize the CTCs and differentiate from leukocytes (i.e., white blood cells) (Bitting et al., 2013).

A new possible way to detect CTCs and monitor them through the bloodstream of PCa patients uses LAPS (Light Addressable Potentiometric Sensor), a multipurpose, biofunctionalised and label-free platform for CTC detection.

Prostate cancer is of epithelial origin and EpCAM is highly expressed in such types of cancer, which allows it to be a good diagnostic marker of PCa CTCs. The LAPS surface is first functionalised with APTES (aminopropyltriethoxysilane) before anti-EpCAM and carboxylated GO are covalently bonded to its surface to form the CTC-LAPS biosensor. Electronic signals from a LAPS are enhanced when GO is attached to its surface (Gu et al., 2015).

This method was tested *in vitro* by using PCa cell lines (LNCaP, C4-2, PC-3) as CTCs and inserting them into Phosphate Buffered Saline (PBS) and healthy human blood. The LAPS output increased with increased with increasing CTC concentration. Characterisation methods applied involved UV-spectrophotometry, scanning electron microscopy (SEM), X-ray photoelectron spectroscopy (XPS) and immunofluorescent assay (IFA). The general CTC-LAPS synthetic methodology is summarised in Figure 13 (Gu et al., 2015).

Carbon nanomaterials have also been reported to be useful to sensitise prostate cancer cells to platinum-based chemotherapeutics, demonstrating that the anti-proliferative and pro-apoptotic effects of two structurally diverse chemotherapeutics, docetaxel (DTX) and mitomycin C (MMC), could be enhanced when administered in combination with carbon nanofibers and carbon nanotubes (Erdmann et al., 2017). This confirms that the local co-administration of chemotherapeutics with carbon nanomaterials could result in a reduction of the chemotherapeutic dosage and thus limit systemic side effects and prevent chemoresistance.

#### 4.2.4 Core-Shell Metallic Nanoparticles for PCa Biosensors

Nanoparticles made up of metal precursors, like gold, copper and silver, are commercially available in a variety of sizes and shapes like rods and dots. Their surface is based in a spherical inner dielectric core such as silica for stability reasons. There is also an outer thin layer coat which consists mainly by a metal. Generally, the properties of these metal based NPs vary accordingly to the ratio of the core and outer coat (Luo et al., 2015). Metallic oxide NPs are also available, varying in their application from ZnO, as a composite in sunscreens to nanosilver particles used in detergents. Gold nanoshells are of great use in nanomedicine since upon arrival to the targeted site, they can efficiently release the drug that they carry by electrostatic stabilization of the drug and the metal outer coat.

Another important aspect of metal nanoshells, is that they can be tagged with a specific antibody or protein that act as biomarkers recognising anomalous tissues. This idea has been further developed in imaging of cancerous cells where the optical properties of NPs are taken into consideration. Clinical trials using superparamagnetic NPs (e.g., MnO) showed a successful drug release by magnetic fields applied to the NP (Sweet et al., 2012; Luo et al., 2015). Argument has been arisen for their use in the human body as they are independent particle that induce diverse responses in the immune system that are harmful. Decreasing size of gold, silver and copper NP was found to be linearly correlated to this toxic effect. Further research is required to overcome this complication and to synthesize effective and safe metal based NPs.

From a bioimaging point of view, current fluorescent labels used in life sciences are based on organic compounds with limited lifetime, which are expensive or toxic and have low kinetic stability in biological environments. To address these challenges, luminescent nanomaterials have been conceived as hierarchical shell-core structures with spherical morphology and tightly controlled dimensions. These tailor-made nanophosphors incorporate rare earths as the active agents in suitable inorganic matrices, such as vanadates, aluminates, yttria fluorides, etc.) (Luo et al., 2011) and are encapsulated in biocompatible shells that isolate the active luminescent core from the medium, avoiding fluorescence decay effects due to interaction with the molecules of the medium (Isasi et al., 2018). On the other hand, this coating allows subsequent functionalisation to provide, for example, selectivity to the final particle to a particular cell line, such as PC-3 (Calatayud et al., 2021).

#### 4.2.5 Liposomes for PCa Biosensors

Nanocarriers (Chen et al., 2016a) have been shown to exhibit coniderabler EPR (Enhanced Permeability and Retention) effects, and so are suitable to carry a range of anti-cancer drugs. Some nanocarrier examples include nanoemulsions, liposomes and polymeric NPs. The multiple binding sites on the nanocarriers’ surface are also used to conjugate molecules so as to actively target cancer cells (Tian et al., 2018). Novel ‘3-in-1’ NPs have also been developed, with the ability to diagnose, image and treat PCa simultaneously (Gindy and Prud’homme, 2009; Kim et al., 2010).

Compared to other types of NPs, liposomes are characterised by a vesicular structure. The vesicle is surrounded by a lipid bilayer made up by artificial or even natural occurring phospholipids, including fatty acids and mono-tri-glycerides. Liposomes are classified into two categories; unilamellar and multilamellar. Mechanistic methods can be used for their preparation, involving the fusion of already existing vesicles or by interchanging organic solvent (Mirzaei et al., 2017).

They are composed of an aqueous inner core, made up of lipophilic molecules and a hydrophobic lipid bilayer on the outside. This lipid bilayer permits them to have a range of functions depending on the size and composition of the lipid vesicle. New strategies are established on liposome technologies to offer a more efficient drug delivery system which will be based on the initiation of the vesicles for targeted drug release. Their outer surface is susceptible to group functionalisation by surfactants, this can further improve their stability for enhanced drug delivery. Over the last decade, biodegradable polyethers, like polyethylene glycol (PGE) were used to improve stability of lipid membranes (Malam et al., 2009). For example, Figure 12B shown the PEG coating’s ability to shields the vesicle and protects it by being removed through the bloodstream in order to be more efficient for active targeting of a specific ligand. Additionally, the structure of the bilayer can easily be fused with other cell membranes due to the similarity of their lipid bilayer. This provides a promising application of NPs to transfer any drug loads that they carry into diseased cells. Liposome technology is continuously emerging as research has proved that there is a limited toxic effect associated with their use. To sum up, the size and composition of the vesicles can determine their applications with the most important existing in the field of biomedicine (Puri et al., 2009).

Recently, elastin-like polypeptide (ELP)-based self-assembling micelles with tethered gastrin-releasing peptide (GRP) on the surface have been suggested to actively target prostate cancer cells. Poorly soluble chemotherapeutics such as docetaxel (DTX) can be loaded into the hydrophobic cores of ELP micelles, but only limited drug retention times have been achieved. Zhang, Wei et al. have reported the generation of hybrid ELP/liposome nanoparticles which self-assembled rapidly in response to temperature change, encapsulated DTX at high concentrations with slow release, displayed the GRP ligand on the surface, and specifically bound to GRP receptor expressing PC-3 cells as demonstrated by flow cytometry. This novel type of drug nanocarrier was successfully used to reduce cell viability of prostate cancer cells *in vitro* through the specific delivery of DTX (Zhang et al., 2018).

Another sample it is reported by De Rosa et al., urotensin-II-targeted liposomes are developed as a new drug delivery system towards prostate and colon cancer cells (Zappavigna et al., 2019). Urotensin-II (UT-II) and its receptor (UTR) are involved in the occurrence of different epithelial cancers. In particular, UTR was found overexpressed on colon, bladder, and prostate cancer cells. The conjugation of ligands, able to specifically bind receptors that are overexpressed on cancer cells, to liposome surface represents an efficient active targeting strategy to enhance selectivity and efficiency of drug delivery systems.

Overall, synthetic nanoplatforms incorporating QDs, AuNPs, liposomes and CPs including GONPs and are just a few examples of nanoparticles used in the sensing and detection of PCa. Chosen for their immense scope of applications and the vast amount of research done on them, they show great potential as breakthrough techniques for early cancer diagnosis.

#### 4.2.6 Deoxyribonucleic Acid-Based Nanoplatforms for PCa Biosensors

Dexosyribonucleic acid has attracted great attention as a nanoplatform due to its predictable secondary structure, small size, high biocompatibility and programmability. DNA strands can be easily engineered into functional nanostructures. These nanoplatforms are compatible with the immune system and can also release pharmaceuticals. As a promising diagnostic and therapeutic nanoplatform, DNA strands combined with other nanoscale materials, such as nanowires, nanotubes, nanosheets, polymers, gold nanoparticles (AuNPs), quantum dots, and iron oxides, show a great potential in early diagnosis of cancer and timely therapy (Chen et al., 2018; Dong et al., 2021). In this context, DNA nanotechnology based on various self-assembled DNA structures for cancer diagnosis and therapy is attracting great interest: DNA origami-based theranostic nanoplatform, DNA hydrogel-based theranostic nanoplatform, DNA signal amplification-based theranostic nanoplatform, DNA-integrated nanomaterials (Chen et al., 2018). Of the latter, the fluorescent nanoparticles integrated with DNA, where upconversion nanoparticles (UCNPs) have been used to facilitate biological detections. Li et al. developed a DNA-driven self-assembled pyramid which was composed of AuNPs and UCNPs. This pyramid could output dual-mode signals to achieve ultrasensitive and highly selective detection of miRNA in live cells (Lin et al., 2017).

## 5 Considerations Towards the Challenges of Prostate *Cancer* Treatment

The main way to assess a potential anticancer drug in order to ensure its safe use in cancer patients and any possible side effects is clinical trials. These consist of four phases (I, II, III and IV), and each has a different purpose in identifying effective drugs. Phase I is concerned with finding the maximum tolerated dose (MTD) of a drug that is safe for a patient to undertake and creating a safety profile for that drug. There are specific rules that must be followed to ensure patient safety during this procedure. Phase II consists of two parts, phase IIA and phase IIB. The first part is concerned with finding whether the drug has the required anti-tumour activity at MTD while the second part is concerned with finding potential agents to send to phase III of the trials (Guan, 2012). Phase III of clinical trials compares the effectiveness of the proposed drug against current treatment and in phase IV the general risks and benefits of the drug are identified after the drug is licenced (Sedgwick, 2011). Clinical trials are essential since they open new pathways for cancer research, in order to improve understanding of the risk it poses and eventually minimise mortality rates and improve the quality of life of cancer patients. Also, when being part of clinical trials, patients avoid the cost of buying their drugs, since they are provided by the research team (Calvin-Lamas et al., 2015). Some reviews even suggest that data from clinical trials should be shared to improve current research (Green et al., 2015; Lo, 2015; Geifman and Butte, 2016), but if this occurs, maximum confidentiality should be undertaken in order to protect the patients’ personal data.

**FIGURE 14:**
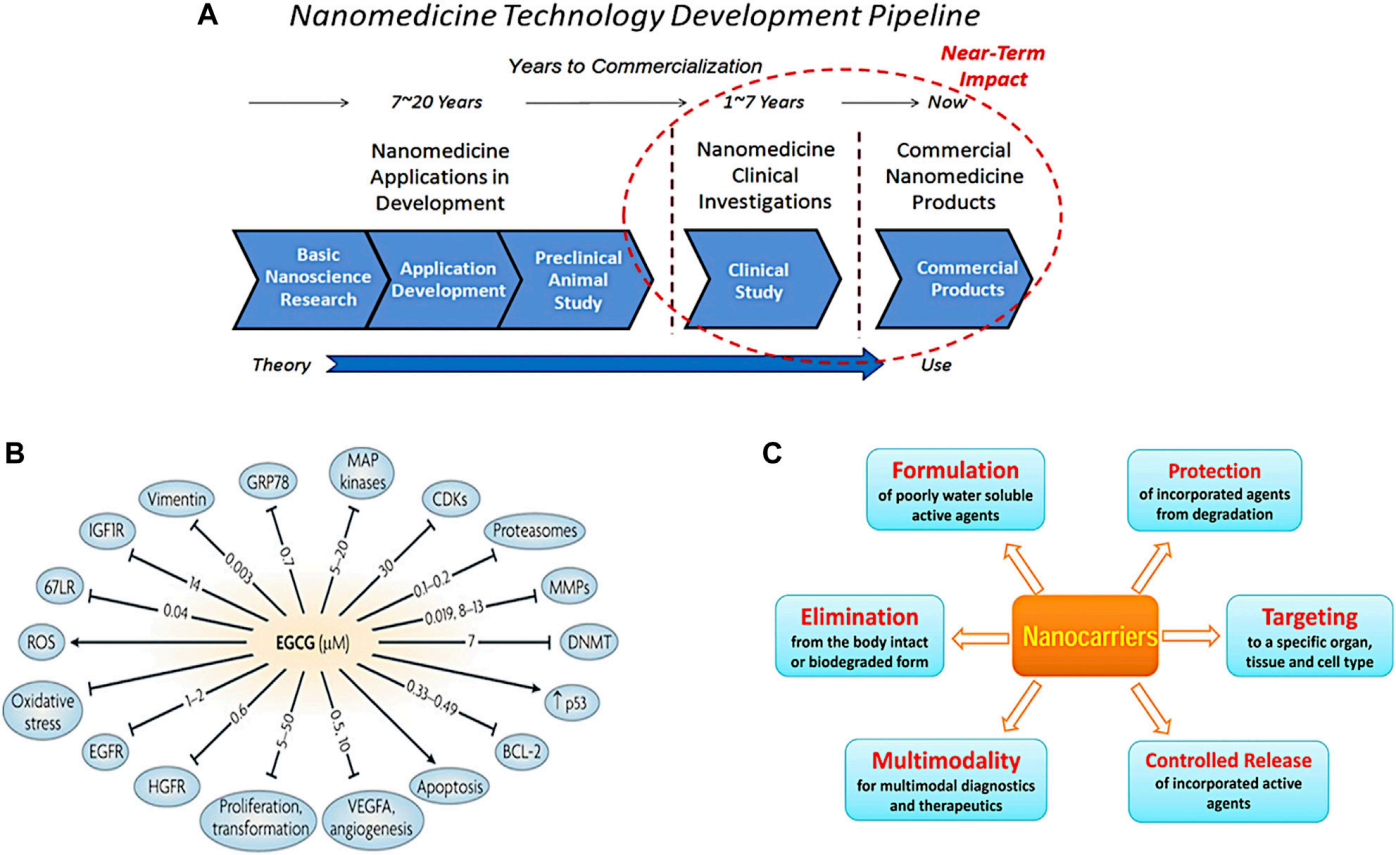
**(A)** The five steps that need to be undertaken before a new nanomedical application is ready for clinical use. (Figure adapted from (Etheridge et al., 2013)), **(B)** possible targets for EGCG (Figure adapted from (Yang et al., 2009)), and **(C)** the advantages of nanocarriers for use in cancer treatment (Figure adapted from (Chen G. et al., 2016)).

The journey any new medicinal method or drug makes until it is deemed safe for use by humans is a complicated and lengthy one. It usually starts in a chemistry lab with some basic science and ends in clinical use. When it comes to a nanomedicine application, this procedure contains five stages which may take several years to complete before a revolutionary nanotechnological treatment is approved for clinical use. Therefore, it will be years before novel techniques and procedures proposed in current literature reviews are put into action (Etheridge et al., 2013). The five stages are outlined in Figure 14A.

As expected, there are certain legal requirements that must be followed during the process of clinical trials, which are designed to promote the scientific integrity of research and take the safety of the participants at high consideration. However, these are costly and may forbid new innovative research. Some are also too time consuming that patient access to new treatments is inexplicably slow (Vose et al., 2017).

Another limiting factor is the low patient participation in clinical (Ahaghotu et al., 2016) which was assigned to socioeconomic or awareness factors, or even eligibility and cultural barriers (Ahaghotu et al., 2016; Kehl et al., 2014; Eggly et al., 2017).

In general, patient participation in clinical trials requires, new, improved and more patient-friendly methods are needed to encourage cancer patient enrolment in clinical trials (Kaplan et al., 2018).

The most common cancer treatments so far involve chemotherapy, radiotherapy and even surgery. Such procedures are harmful for patients in more ways than one. They kill healthy cells as well as cancerous ones and some tumors become immune to the drugs after some time. Also, the side effects can be very harsh on the patients’ health and morale (Magge and DeAngelis, 2015). It is therefore a widespread opinion that the world is in need of new, revolutionary cancer treatments that actively or passively target cancer cells and minimize the chances of reoccurrence (Peer et al., 2007). Nanotechnology and nanomedicine have come a long way in the last 30 years (Peer et al., 2007) and many new ways of cancer treatment using nanoparticles have been proposed. In fact, many of the examples given above for the sensing of PCa have the potential to act as drug carriers as well. The challenge, however, is that only a few of the proposed nanocarriers actually pass through the clinical trials process and are approved for clinical use (Peer et al., 2007). Theranostics are currently being given much attention due to their potential for the development of personalized medicine. NPs form the basis of theranostics, which have vast applications in cancer treatment, not only limited to PCa, but also applying to breast, esophageal and other cancers (Lymperopoulos et al., 2017). Derivatives of green tea have also shown results that would exceed expectations when it comes to treating PCa. Specifically EGCG (epigallocatechin 3-gallate) has shown *in-vitro* anticancer activity with no effect on healthy cells (see Figure 14B) although results appeared to be less promising when tested *in vivo* (Yang et al., 2009; Johnson et al., 2010; Tian et al., 2018).

### 5.1 Considerations for the Future of Nanomedicine for PCa

Several societal concerns that arise with respect to the nanotechnological approaches to PCa diagnosis are highlighted hereby. During the past 2 decades, nanomedicine has gained significant research and societal attention including due to its potential to act as an effective tool in addressing unmet clinical needs in cancer diagnosis and therapy (Etheridge et al., 2013). However, only a small proportion of cancer research involving nanomedicines has led to approvals for their clinical use. This is likely due to the extensive process of clinical trials and legal requirements that must be met before a new drug or medical procedure is approved for clinical use and also to the fact that growth in the medical - and in fact any–industry depends heavily on the state of the economy at the time (Etheridge et al., 2013). The large investments made by pharmaceutical companies and industries on nanomedicine research and development shows that NPs are a very promising tool for future medicine and also that the extent to which cancer affects modern society is fully understood.

On the other hand, even though NPs emerged as promising as theranostic tools against cancer, there are concerns about their behaviour *in vivo*. While most common anti-cancer drugs in clinical use are metabolized and excreted soon after administration, NPs have demonstrated persistence in the body for months or even years (Etheridge et al., 2013). Cadmium-containing QDs for example, are suspected of being toxic and harmful to organic frameworks and are deadly to cells when illuminated with UV-light (Shanker, 2015).

Often the approach to gathering and using relevant information on potential applications creates barriers to medical innovations due to the extensive regulatory and legal procedures that are necessary. For this reason, and according to Etheridge et al. (2013), a clear definition is needed for any regulatory approach to nanomedicine to succeed.

The questions that have arisen concerning the suitability of NPs in medical applications must be addressed by continuous evaluations, according to Chenel et al. (2015). The current measures concentrate on predictions of whether the new applications will be accepted and payed for by those who will use them. These involve predicting the risk of death or injury that the new method(s) poses on the targeted users. The opinion of the patients and their families is seldom taken into account, even though it is of great importance. The patients may not accept the new treatment and essentially refuse to have it used in their body. As a result, the Ethical, Legal and Social Implications (ELSI) raised by new nanotechnologies must be taken into account instead of researchers trying to shape the public’s opinions regarding NPs (Chenel et al., 2015) as well as patients acceptability of new nanomedicines for treatments or taking part in clinical trials (Ahaghotu et al., 2016; Sarkar et al., 2015)

Because the early diagnosis of prostate cancer (PCa) is one of the greatest concerns for ageing men worldwide, the development of fast reliable methods for detecting PCa by electrochemical or optical biosensing is needed to address the unmet need of early diagnosis and monitoring non-invasively the response of PCa to treatment. New biosensing devices leading to tests which will be validated by coordination with imaging scans in a clinical setting and better therapies are needed. Such tests could offer an earlier, and better, diagnostic test for the disease stage discrimination which may help to ensure patients receive the optimum treatment. In addition, new, more selective and specific drugs can be designed thanks to nanotechnology, which will allow more effective and personalized therapies.

The development of nanomaterials for theranostic purposes must consider the potential difficulties that may arise in relation to coordination chemistry, synthesis and encapsulation protocols. New theranostic nanomaterials must exhibit good compatibility between the core, targeting agent, diagnostic moiety and active drug. In addition, the synthesis protocol for these materials should have as few steps as possible, affordable and realistic costs, high reproducibility and ease of scale-up, and should provide diagnostic and therapeutic efficacy. Targeted imaging agents can improve the sensitivity and accuracy of both treatment and diagnostic results by providing real-time monitoring. In this regard, activatable prodrugs can also be applied for real-time monitoring of cancer theranostics, thus aiding in the decision-making process on the most effective therapeutic approach for each patient.

A variety of imaging techniques can be applied for theranostic purposes, i.e., fluorescence imaging, tomography (PET or SPECT), MRI, ultrasound, imaging and more than one technique can be combined to increase the efficacy of the nanomaterials. These are the principles that should govern the development of the new nanotheranostic platforms. However, there are still a number of issues that need to be addressed from our basic understanding of the biology of specific diseases and the biological interactions of the synthetic building blocks involved in the nanoplaforms assemblies in patients, to commercialization hurdles related to manufacturing, costs, and regulatory standards. In this sense, reducing complexity to the minimum required for pathophysiological or medical need is paramount in nanoparticle design and synthesis to generate clinically translatable nanosized therapeutics (Hua et al., 2018).

## 6 Conclusion

Nanotechnology is becoming a highly developed platform for innovative and groundbreaking studies. Increased research efforts have resulted to the introduction of a targeted theragnostic agents that consist of NPs enabling the diagnosis and therapy of various diseases simultaneously. Their easily modified surface can ensure the accommodation of biomolecules and therefore enhance their ability to target a specific site of reaction to be studied. Using such nanoplatforms may contribute to an effective cancer molecular imaging and treatment. A variety of innovations in nanotechnology applications towards sensing and imaging of PCa focuses on target identification, bioassay development and quality control for early detection of PCa using nanotechnology. However, there remains an ongoing debate regarding the long-term suitability of these methods, with the society expressing concerns about possible toxicity resulting from the NPs’ persistence in living systems.

Addressing challenges in nanomedicines design would become of interest for a number of sectors, in particular the healthcare industry and the electronics sector, where there is a continuing need for miniaturization and circuit integration of electronic devices with cellular environments and biological materials, also by nurses and clinicians that may be using the technology. This will have clear benefits to individuals, society as a whole and a tangible economic impact as new biosensing technology, underpinned by clinical radiology imaging could emerge, however the combined advantages of these diagnostic tools would need to be validated in clinical settings.

Development of fast and reliable methods for detecting PCa by electrochemical or optical biosensing would enable both early diagnosis and non-invasive post-diagnosis monitoring. Current research challenges focus on target identification, bioassay development and quality control for early detection of PCa using nanotechnology. This will have an impact socially and economically. The patients and their carers will be the main beneficiaries. The innovative approaches developed through our research will lead to significant improvements in the ability of clinicians to detect and treat prostate cancer early before the appearance of extensive tumour symptoms and will improve the monitoring of prostate cancer as well as its disease progression in clinical trials. Other key potential stakeholders who can benefit from theanostic approaches to the early PCa detection include public sector organisations, industry, third sector organisations, researchers, charities, patient focused groups and the public. From and economical point of view there is a worldwide drive towards novel biosensor technologies for biomedical applications including drugs/biomarkers detection in living systems. The Global Biosensors Market size is forecasted to reach $34.3 billion by 2025 yet design and development challenges remain towards achieving simpler and more accessible biosensors to analyse biomolecular interactions in real time, for new applications in drug discovery, including target identification, ligand recognition assays, assay development and quality control.
