# Corrigendum: Nano-Theranostics for the Sensing, Imaging and Therapy of Prostate Cancers

**DOI:** 10.3389/fchem.2022.919505

**Published:** 2022-05-11

**Authors:** David G. Calatayud, Sotia Neophytou, Eleni Nicodemou, S. Giuseppe Giuffrida, Haobo Ge, Sofia I. Pascu

**Affiliations:** ^1^ Department of Chemistry, University of Bath, Bath, United Kingdom; ^2^ Department of Electroceramics, Instituto de Ceramica y Vidrio - CSIC, Madrid, Spain; ^3^ Centre of Therapeutic Innovations, University of Bath, Bath, United Kingdom

**Keywords:** nanomedicine, theranostics, sensing, imaging, therapy, prostate cancer

In the original article, in the **Funding** statement on page 29, the authors regretably omitted to include the funder “Cancer Research at Bath (CR@B) and membership of the Centre of Therapeutic Innovation at University of Bath for author SIP.” The correct **Funding** statement is as follows:

“SIP acknowledges funding from ERC Consolidator Grant O2Sense 617107 (2014–2020) and ERC Proof of Concept Grant Tools-To-Sense 963937 (2020–2022), EPSRC (EP/K017160/1 “New manufacturable approaches to the deposition and patterning of graphene materials”), Innovate United Kingdom (previously Technology Strategy Board-CR&D, TS/K001035/1), STFC, University of Bath (UoB) Impact fund, EPSRC Centre for Doctoral Training Centre for Sustainable Chemical Technologies (EP/G03768X/1), Cancer Research at Bath (CR@B) and membership of the Centre of Therapeutic Innovation at University of Bath. DGC also thanks Fundación General CSIC (COMFUTURO Program) for funding.”

In the original article, there was a minor error in the caption for Figure 1, page 5, as published. The captions for parts (A) and (B) were incorrect. The correct caption is given below.

**FIGURE 1:**
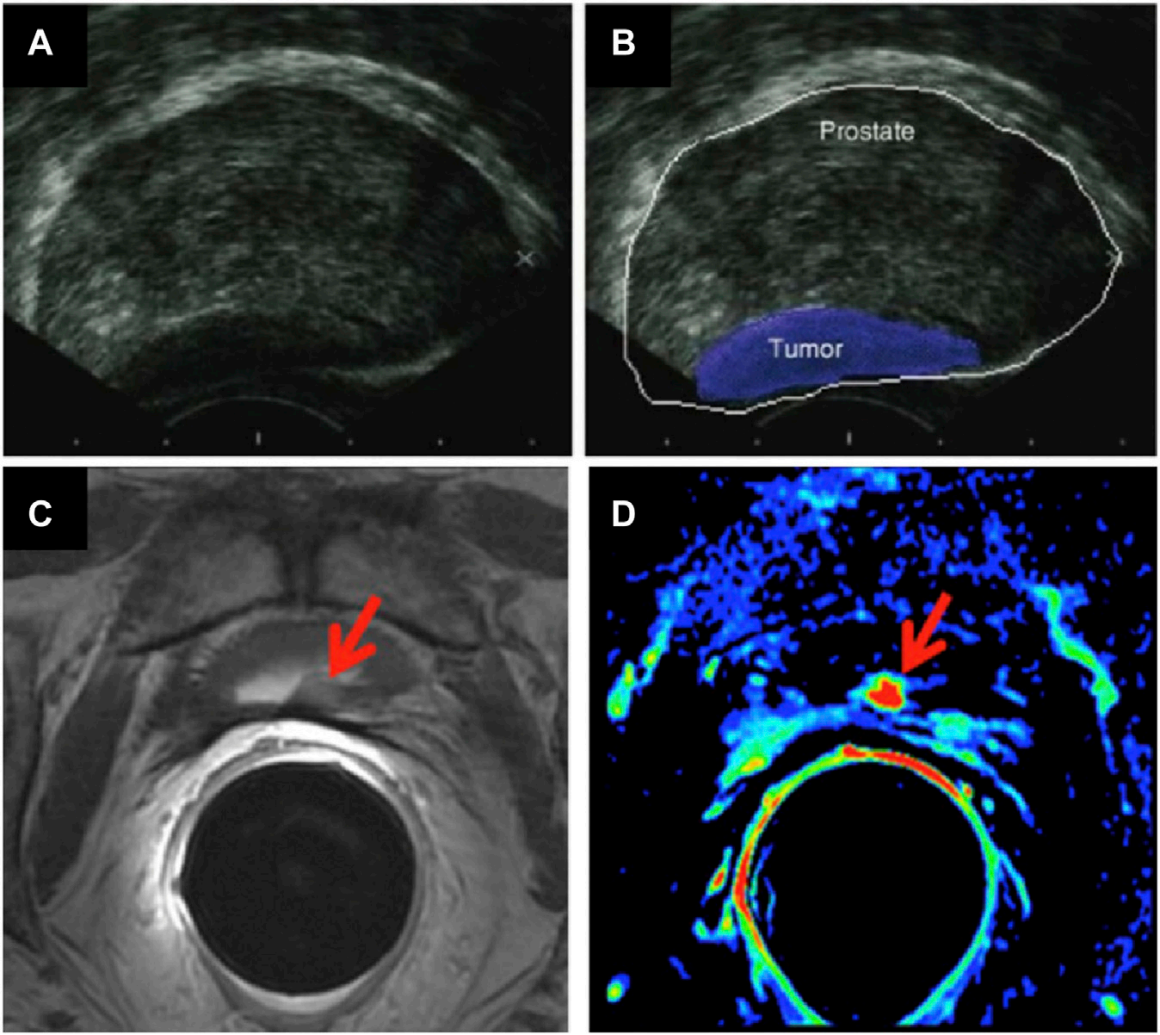
**(A,B)** TRUS images of a prostate affected by cancer (figure adapted from (Theodorescu and Ehdaie, 2009)); and MRI images of the prostate gland, showing cancerous regions (arrows): **(C)** Axial T2-weighted fast spin-echo image and **(D)** axial gradient-echo T1-weighted colour map image (figure adapted from (**Maurer et al., 2016**)).”

In the original article, there was a minor error in the caption for Figure 12, page 23, as published. The captions for parts (B) and (C) were incorrect. The correct caption is given below.

**FIGURE 12:**
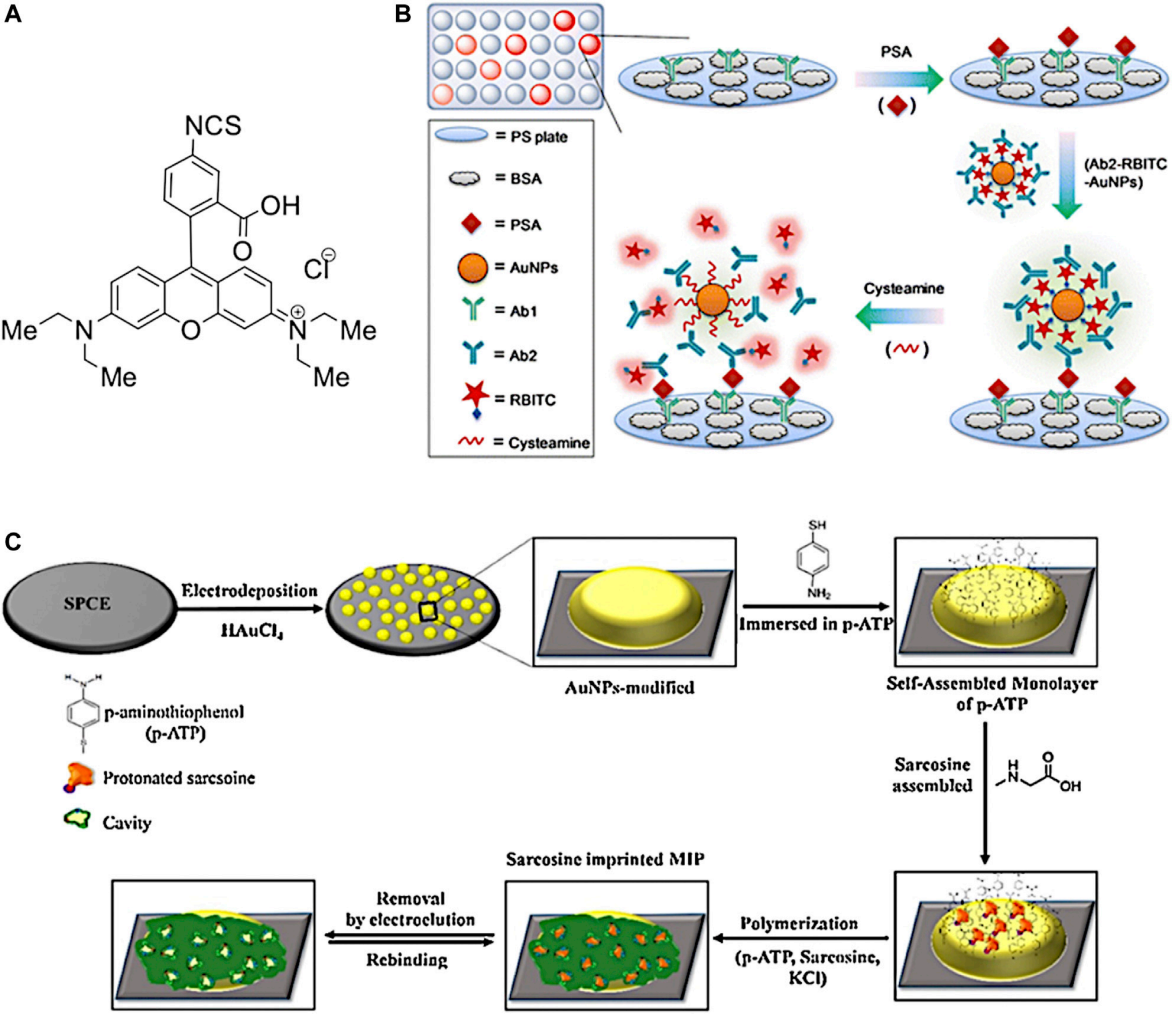
**(A)** Example of a fluorescent dye (denoted RBITC) used in the synthesis of the fluorescence-activated probe Ab2-RBITC-AuNP, **(B)** procedure as demonstrated by D. Liu et al. for the synthesis of the AuNP activated probe for early PSA detection (figure adapted from Ref (Liu et al., 2013)) **(C)** an overview of the procedure in described for the formation of MIP/AuNPs/SPCE (figure adapted from (Nguy et al., 2017)).

In the original article, there was an error regarding the affiliation for author “Sofia I. Pascu.” As well as affiliation 1, the author is also affiliated with the following institution:

“Centre of Therapeutic Innovations, University of Bath, Bath, United Kingdom.”

In the original article, “Cancer Statistics, 2021” was not cited. The citation belongs in the **Introduction**, page 2, paragraph 4, and the corrected sentence reads as follows:

“On average, the 5-years survival rate of patients with localized PCa exceeds 90%. However, patients with distant metastases have significantly lower 5-years survival rates, averaging approximately 31% for prostate cancers (Siegel et al., 2021).”

In the original article, some references were missing. The additional references are listed below.

The authors apologize for these errors and state that this does not change the scientific conclusions of the article in any way. The original article has been updated.
